# A novel dual-prodrug carried by cyclodextrin inclusion complex for the targeting treatment of colon cancer

**DOI:** 10.1186/s12951-021-01064-3

**Published:** 2021-10-19

**Authors:** Lin Chen, Yan Lin, Zijun Zhang, Ruisheng Yang, Xiaosheng Bai, Zhongbing Liu, Zhongling Luo, Meiling Zhou, Zhirong Zhong

**Affiliations:** 1 Department of Pharmaceutical Sciences, School of Pharmacy, Southwest Medical University, Luzhou, 646000 Sichuan China; 2 Nanchong Key Laboratory of Individualized Drug Therapy, Department of Pharmacy, Nanchong Central Hospital, The Second Clinical Medical College, North Sichuan Medical College, Nanchong, 637000 Sichuan China; 3 Department of Pharmacy, the Affiliated Hospital of Southwest Medical University, Luzhou, 646000 Sichuan China

**Keywords:** Butyric acid, 5-Aminosalicylic acid, Prodrug, Inclusion complex, Colon cancer

## Abstract

**Background:**

There is an obvious correlation between ulcerative colitis and colorectal cancer, and the risk of colorectal cancer in patients with ulcerative colitis is increasing. Therefore, the combination therapy of anti-inflammatory and anti-tumor drugs may show promising to inhibit colon cancer. 5-aminosalicylic acid (5-ASA) with anti-inflammatory function is effective for maintaining remission in patients with ulcerative colitis and may also reduce colorectal cancer risk. Histone deacetylase (HDAC) plays an essential role in the progression of colon cancer. Butyric acid (BA) is a kind of HDAC inhibitor and thus shows tumor suppression to colon cancer. However, the volatile and corrosive nature of BA presents challenges in practical application. In addition, its clinical application is limited due to its non-targeting ability and low bioavailability. We aimed to synthesize a novel dual-prodrug of 5-ASA and BA, referred as BBA, to synergistically inhibit colon cancer. Further, based on the fact that folate receptor (FR) is over-expressed in most solid tumors and it has been identified to be a cancer stem cell surface marker in colon cancer, we took folate as the targeting ligand and used carboxymethyl-β-cyclodextrin (CM-β-CD) to carry BBA and thus prepared a novel inclusion complex of BBA/FA-PEG-CM-β-CD.

**Results:**

It was found that BBA/FA-PEG-CM-β-CD showed significant inhibition in cell proliferation against colon cancer cells SW620. It showed a pro-longed in vivo circulation and mainly accumulated in tumor tissue. More importantly, BBA/FA-PEG-CM-β-CD gave great tumor suppression effect against nude mice bearing SW620 xenografts.

**Conclusions:**

Therefore, BBA/FA-PEG-CM-β-CD may have clinical potential in colon cancer therapy.

**Graphical Abstract:**

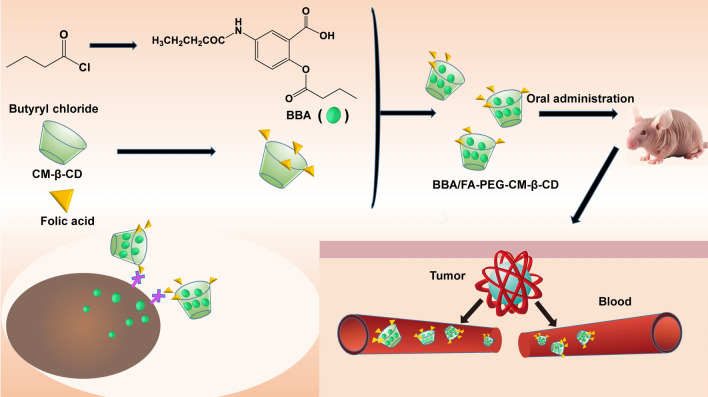

**Supplementary Information:**

The online version contains supplementary material available at 10.1186/s12951-021-01064-3.

## Introduction

Colon cancer is a common malignant tumor of the digestive tract that tends to occur at the junction of the rectum and sigmoid colon. The pathogenesis of colonic cancer is concerned with living habits, genetic factors, and inflammatory bowel disease [1, 2]. The abnormal proliferation of colon cancer cells is closely related to the abnormal transcription of specific genes. Histone acetylation and deacetylation of chromatin are some of the key steps in regulating gene transcription [3]. The histone acylation state is coordinated and controlled by histone acetylases (HAT) and histone deacetylases (HDAC), which mediate nucleosome structural changes and regulate gene transcription, and participate in cell cycle progression and differentiation [4].

At present, the treatment of colon cancer mainly includes surgery and drug treatment [5, 6]. The conventional drugs for the treatment of colon cancer mainly include 5-fluorouracil (5-FU) [7], capecitabine, tigafluorin, irinotecan, and oxaliplatin [8]. However, tumor cells have developed resistance to these chemotherapeutic drugs [9, 10]. In addition, the side effects also limit the maximum allowable amount, resulting in a limited amount of drug accumulated in tumor tissue that is insufficient to reach the effective therapeutic concentration [11, 12]. Studies have shown an obvious correlation between ulcerative colitis and colorectal cancer, and the risk of colorectal cancer in patients with ulcerative colitis is increasing [13, 14]. High levels of pro-inflammatory cytokines such as IL-1β are closely associated with TNF-α in colorectal cancer formation [15]. Therefore, we took the combination therapy of anti-tumor drug and anti-inflammatory drug to inhibit synergistically colon cancer and improve the therapeutic efficacy [16].

It was reported that 5-ASA could inhibit NF-κB and scavenges free radicals to treat inflammation [17]. It is effective for maintaining remission in patients with ulcerative colitis and may also reduce colorectal cancer risk. Its derivative 2-hydroxyl-5-butylaminobenzoic was reported to show an anti-inflammatory effect on acetic acid-induced colitis in rats [18]. Butyric acid (BA) was found to inhibit Cox-2 activation through HDAC, which induced apoptosis of colon cancer cells [19]. BA could also inhibit histone deacetylation, promote histone hyperacetylation, reduce DNA transcriptional activity, and inhibit colon cancer cell proliferation [20, 21]. But BA is weakly acidic, easy to volatilize, and shows corrosive to the human body. The clinical application of BA is limited due to its non-targeting ability and low bioavailability. Abraham Nudelman has shown that the one kind of prodrugs of butyric acid increased aqueous solubility and potential for treating cancer [22]. Therefore, in this study, we planned to synthesize a novel dual-prodrug of BA and 5-ASA, 2-butyryl oxy-5-butylaminyl benzoic acid (BBA), combining the anti-tumor role of BA and anti-inflammatory function of 5-ASA or its derivative.

The poor pharmacokinetic characteristics of BBA may limit its application with the traditional dosage form. It was reported that cyclodextrins (CDs) could be used as drug carrier due to the particularity of the cavity [22, 23], which showed many advantages such as extending blood circulation time, improving the solubility of hydrophobic drugs, controlling drug release mode, and protecting drug degradation in vivo [24]. Among many kinds of cyclodextrins, carboxymethyl (CM-β-CD) could be used in drug extended-release dosage forms and showed lower hemolysis than β-CD, γ-CD, or HP-β-CD [25]. Therefore, CM-β-CD was selected as the drug carrier in this study.

Moreover, some studies have shown that cyclodextrins are known to be barely hydrolyzed and only slightly absorbed in passage through the stomach and small intestine. This unique property makes cyclodextrins useful as a targeting carrier to the colon.

A folic acid receptor is a glycoinositol-conjugated protein that is highly expressed in a variety of tumor cells, including ovarian, colon, liver, kidney, lung, and breast epithelial malignancies [26–28]. Folic acid (FA) is widely used in cancer treatment. Compared with other ligands, FA exhibits some advantages of high affinity for the receptor, obvious specificity, good coupling, low cost, and non-immunogenicity [29, 30]. In this experiment, folic acid was used as a ligand to modify CM-β-CD, further improving the drug targeting, reducing the side effects of drugs, playing a better curative effect, and achieving the purpose of tumor suppression [31].

Taking together, this project firstly intended to synthesize the dual-prodrug BBA of 5-ASA and BA through the two-step chemical reaction. Then taking the functional PEG as the coupling agent, FA was connected to CM-β-CD through the amide reaction principle. The novel inclusion complex of BBA/FA-PEG-CM-β-CD was prepared by a saturated aqueous solution method. MTT assay was used to determine the inhibitory effect of BBA/FA-PEG-CM-β-CD on cell proliferation. Flow cytometry was taken to detect the cell cycle and cell apoptosis. Further, we established the colon cancer model in BALB/C nude mice, performed real-time fluorescence imaging analysis to detect the distribution of BBA/FA-PEG-CM-β-CD in vivo, and conducted in vivo pharmacodynamics detection in nude mice to evaluate the tumor inhibitory effect of BBA/FA-PEG-CM-β-CD.

## Methods

### Materials

5-ASA was provided by Runze Bentu Chemical Co., Ltd (Chengdu, China). CM-β-CD was from Solarbio Life Sciences (Beijing, China). Butyryl chloride, butyric anhydride. FA-PEG_2000_-NH_2_ and coumarin-6 were provided by Ruixi Biotechnology (Xi’an, China). DID was obtained from Keygen Biotechnology (Nanjing, China) and 5-fluorouracil from Tianjin Jinyao Medicine Pharmaceutical Co., LTD (Tianjin, China).

Dulbecco’s Modified Eagle’s Medium (DMEM), streptomycin, and penicillin were bought from Hammer Flew Biochemical (Beijing, China). Fetal bovine serum (FBS) was provided by TianHang Biotechnology (Huzhou, China), and thiazolyl blue tetrazolium bromide (MTT) was bought from Beyotime Biotechnology (Shanghai, China).

### Cell lines and animals

BALB/C nude mice (18–25 g) aged 4–6 weeks were from SPF Biotechnology Co, Ltd (Beijing, China, certificate number SCXK2019-0010). Male SPF Kunming mice (19 ± 25 g) were provided by Dashuo Experimental Animal Company (Chengdu, China, SCXK2020-303). All experimental procedures were approved by the Animal Ethics Committee of Southwest Medical University (No. 20200010) and were performed in strict accordance with the regulations of the Animal Protection and Use Committee of Southwest Medical University.

The human colon adenocarcinoma cell line CaCo-2, and human colon cancer SW620 cells were purchased from the Shanghai Cell Institute of the Chinese Academy of Sciences. CaCo-2 and SW620 cells were cultured in DMEM containing 10% FBS, 100 U/mL penicillin, and 100 mg/mL streptomycin at 37 °C with 5% CO_2_.

### Synthesis of BBA and FA-PEG-CM-β-CD

**Fig. 1:**
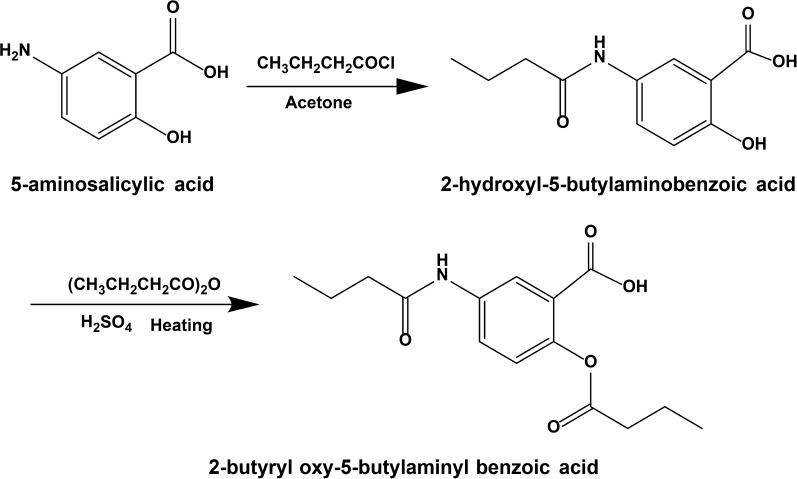
Synthesis of 2-butyryl oxy-5-butylaminyl benzoic acid (BBA) as the dual-prodrug of butyric acid (BA) and 5-aminosalicylic acid (5-ASA)

The dual-prodrug of 2-butyryl oxy-5-butylaminyl benzoic acid (BBA) was synthesized as shown in Fig. 1.

Firstly, it is the synthesis of 2-Hydroxyl-5-Butylamino Benzoic Acid. To 5-aminosalicylic acid (1.31 mmol) in NaHCO_3_ solution (16 mL) saturated with CO_2_, a solution of butyryl chloride (0.27 mL) in acetone was added slowly at 0 °C, then the mixture was stirred at room temperature for 4 h [32]. After evaporation of the solvents under vacuum, the product was cooled to 0 °C, then acidified by adding hydrochloric acid (0.5 mL). The last product of 2-hydroxyl-5-butylamino benzoic acid was filtered, freeze-dried, and stored at − 20 °C [18].

Secondly, 2-hydroxyl-5-butylaminobenzoic acid (0.546 mmol) was added to butyric anhydride, and a few drops of concentrated sulfuric acid were added slowly [33]. After stirring at 80 °C for 5 h, the product was added to ice water (150 mL) and incubated for 2 h. After the crystal was completely precipitated out, the product was filtered-dried in a vacuum for 12 h to get BBA.

To prepare FA-PEG-CM-β-CD, N-hydroxysuccinimide (NHS, 0.0122 mmol) and 1-ethyl-3-(3-dimethylaminopropyl) carbodiimide hydrochloride (EDC, 0.0224 mmol) were mixed with CM-β-CD (3 mL, 0.012 mmol) in ultra-pure water to activate the carboxyl group of CM-β-CD and stirred for 15 min. Then FA-PEG_2000_-NH_2_ (0.01 mmol) was added into the mixture and stirred at room temperature for 24 h in a dark room, dialyzed against water for 10 h, and freeze-dried to obtain FA-PEG-CM-β-CD [31].

### Preparation of inclusion complex

The inclusion complexes of BBA/CM-β-CD or BBA/FA-PEG-CM-β-CD were prepared by the saturated aqueous solution method at 1:2 molar ratio of BBA and CM-β-CD or FA-PEG-CM-β-CD. Briefly, BBA/CM-β-CD or FA-PEG-CM-β-CD dissolved in ultra-pure water (4 mL) was mixed with BBA which was dissolved in methanol, and sonicated for 40 min at 40 °C. After evaporation of the solvent at 35 °C, the product was freeze-dried to obtain BBA/CM-β-CD or BBA/FA-PEG-CM-β-CD [34, 35].

### Characterization of inclusion complex

Fourier transformed infrared (FTIR, IRAffinity-1S, Shimadzu, Japan) was applied to analyze the formation of BBA/FA-PEG-CM-β-CD. Briefly, the specimens were blended with KBr at a ratio of 1:100 (w/w), taking BBA, blank inclusion complex of FA-PEG-CM-β-CD, and the physical mixture of BBA with FA-PEG-CM-β-CD as the controls. Then, KBr discs were prepared under 10,000 psi hydraulic pressure. FTIR spectra were carried out with a resolution of 4 mm/s on KBr pellets with a wavenumber range of 500–4000 cm^−1^.

X-ray diffraction (XRD) analysis was performed for BBA, FA-PEG-CM-β-CD, BBA/FA-PEG-CM-β-CD using X-ray powder diffractometer (XRD, BRUCKER D8 ADVANCED, Bruker, Germany), where Cu Kα radiation was applied in the 2θ range of 3° − 50°. Thermal gravimetric analysis (TGA) measurements and differential scanning calorimetry (DSC) analysis were performed for the inclusion complex under nitrogen atmosphere by heating the samples at the rate of 10 °C/min from 30 °C up to 600 °C by the synchronous thermal analyzer (TG-DSC, STA 449 F5/F3 Jupiter, NETZSCH, Germany) [36]. Morphological characterization of BBA, FA-PEG-CM-β-CD, BBA/FA-PEG-CM-β-CD was examined using scanning electron microscopy (SEM, Hysitron TI 950, USA).

To get the encapsulation efficiency (EE, %) and drug-loading rate (DL, %), BBA was detected by the high-performance liquid chromatography (HPLC) analysis. Briefly, BBA was quantified on an Agilent ZORBAXSB-C18 column (4.6 × 150 mm, 5 µm) at 30 °C attached to an Agilent 1260 HPLC system (Infinity, USA). The mobile phase was mixture of acetic acid (0.1 mol/L) and acetonitrile (v: v = 50: 50) at a flow rate of 1.0 mL/min. It was monitored at 240 nm, and the injection volume was 20 µL. Moreover, the chromatographic conditions were validated through specificity, linearity, precision, and blank recovery. EE and DL were calculated using the formula:

$${\text{EE }}\left( \% \right) \, = \, \left( {{\text{M}}_{{{\text{total}}}} - {\text{M}}_{{{\text{free}}}} } \right) \, /{\text{ M}}_{{{\text{total}}}} \times { 1}00\%$$

$${\text{DL }}\left( {{\text{mg}}/{\text{g}}} \right) \, = \, \left( {{\text{M}}_{{{\text{total}}}} - {\text{M}}_{{{\text{free}}}} } \right)/{\text{M}}_{{\text{inclusion complex}}}$$

Release of BBA from BBA/CM-β-CD and BBA/FA-PEG-CM-β-CD was investigated in vitro using the dialysis method. Briefly, 1 mL of BBA/CM-β-CD, BBA/FA-PEG-CM-β-CD, and free BBA were respectively placed into dialysis bags with a molecular weight cut-off of 3 kDa (Spectrum-labs, USA) and dialyzed at 37 °C against phosphate-buffered saline (PBS) containing 0.5% (w/v) Tween 80 at pH 7.4 with gentle stirring [37]. At different time points (0.25, 0.5, 1, 2, 3, 4, 6, 8, 12, 48, 72 and 96 h), 1.0 mL of release medium was removed and replaced with fresh medium. The sample was filtered by a 0.22 μm filter membrane, and the filtrate was used to detect BBA by HPLC as described above, and experiments were performed three times [38].

To evaluate the stability, BBA/FA-PEG-CM-β-CD was placed respectively at high temperature and high humidity conditions. For the high-temperature experiment, the inclusion complex of BBA/FA-PEG-CM-β-CD (50 mg) was placed in a weighing flask at 60 °C for 10 days. The samples were taken respectively at 0 d, 5 d, and 10 d to detect the encapsulation efficiency. If the content of effective ingredients decreased by 5%, the same operation was further conducted at 40 °C.

For the high humidity conditions experiment, BBA/FA-PEG-CM-β-CD inclusion complex (50 mg) was placed openly in a closed container (25 °C) with a relative humidity of 75% ± 5% and 95% ± 5% for 10 d, in which NaCl and KNO_3_ saturated solution were respectively placed in the closed container. The samples were taken respectively at 0 d, 5 d, and 10 d to detect the encapsulation efficiency [39, 40].

### Anticancer activity of BBA/FA-PEG-CM-β-CD

CaCo-2 and SW620 cells were used as model cells to evaluate the anticancer effects in vitro. Single-cell suspension was prepared with DMEM containing 10% Gibco FBS and 1% penicillin–streptomycin. The cells were seeded in a 96-well plate (3500 cells/well) and incubated respectively for 24 h, 48 h, and 72 h at 37 °C with 5% CO_2_. BBA, FA-PEG-CM-β-CD, BBA/CM-β-CD, BBA/FA-PEG-CM-β-CD, as well as sodium butyrate (NaB), 5-ASA, the physical mixture of NaB and 5-ASA (NaB + 5-ASA), and the positive control of 5-FU were added to the wells at different concentrations and incubated for 24 h, 48 h and 72 h at 37 °C with 5% CO_2_. The medium was removed, and cells were washed three times with PBS before incubation with MTT for 4 h at 37 °C. After the medium was removed, 100 μL of DMSO was added to the well for 10 min. The absorbance of the solution was measured at 570 nm by Multimode Reader (Bio Tek Synergy H1) [41]. The cell-growth inhibition rate was calculated as the formula:

$${\text{Cell growth inhibition rate }}\left( \% \right) \, = { 1}00 \, {-} \, \left( {{\text{A}}_{{{\text{sample}}}} - {\text{A}}_{{{\text{blank}}}} } \right) \, / \, \left( {{\text{A}}_{{{\text{control}}}} - {\text{A}}_{{{\text{blank}}}} } \right) \, \times { 1}00$$

### Cellular uptake of inclusion complex

As a drug delivery system, whether BBA/FA-PEG-CM-β-CD inclusion complex could deliver drugs to target cells, is an important index to evaluate the targeting of the drug delivery system. In this study, CaCo-2 cells and SW620 cells as cell models were used to investigate the cell uptake of the inclusion complex. Coumarin 6 (20 μg/kg) was used together with BBA to prepare BBA/CM-β-CD (30 μM) and BBA/FA-PEG-CM-β-CD (30 μM), which could emit green fluorescence. CaCo-2 cells and SW620 cells in good growth condition and logarithmic growth phase were plated on the sterile coverslips (3 × 10^5^ cells/well) in 24-well plates, and cultured for 24 h. After the original medium was removed, 1 mL of BBA/FA-PEG-CM-β-CD and BBA/CM-β-CD diluent were added separately, placed in an incubator for 2 h. Additionnally, both CaCo-2 cells and SW620 cells were treated with BBA/FA-PEG-CM-β-CD in blank medium containing different concentrations of free folic acid (FA). After incubation, the culture medium was removed, washed thoroughly twice with PBS, and fixed in 4% paraformaldehyde for 30 min, and then staining was performed with 150 μL of DAPI (5 mg/mL) for 5 min. Glycerol was used to seal coverslips, and a confocal laser-scanning microscope was utilized to observe them at an emission wavelength of 477 nm. To quantify cellular uptake, Image J (NIH Image-Pro Plus 6.0) was used [42].

### Cell apoptosis and cell cycle analysis

SW620 cells were taken as the cell model to investigate the cell apoptosis and cell cycle effects of BBA/FA-PEG-CM-β-CD by flow cytometry technology. SW620 cells were seeded in the 6-well plates (3 × 10^6^ cells/well) and BBA (30 μM), BBA/FA-PEG-CM-β-CD (30 μM), and BBA/CM-β-CD (30 μM) was added to the well and incubated for 48 h at 37 °C with 5% CO_2_.

For the cell apoptosis detection, the cells in the different groups were washed three times with PBS and incubated with Annexin V-FITC solution for 10 min. Then, flow cytometry analysis was performed to detect the FITC and PI fluorescent intensity and report the percentage of the cell population within each plot quadrant [43].

For the cell cycle analysis, the cells in the different groups were washed three times with PBS and incubated with 1 mL of pre-cooled 70% alcohol at 4 °C for 24 h. After the cells were fixed, centrifuged at 300 × g for 5 min to collect the cells. 1 mL of cold PBS was added to re-suspend the cells and centrifuged at 300 × g for 5 min to remove the supernatant. 10 μL of RNaseA (50 ×) and 25 μL of propidium iodide (20 ×) in 0.5 mL of buffer were added [44]. Finally, flow cytometry was used for cell cycle detection.

### Acute toxicity assessment of BBA/FA-PEG-CM-β-CD in mice

To determine the potential toxicity in vivo and guide the analysis of anti-tumor efficacy in vivo, an acute toxicity study of BBA/FA-PEG-CM-β-CD was conducted in Kunming mice by a single oral administration of the tested formulations, and the treated mice were observed for 14 d.

After fed adaptively for 5 d, the animals were randomly divided into 8 groups (n = 5) following as NaB (2.5 mmol/kg), 5-ASA (1.25 mmol/kg), FA-PEG-CM-β-CD (0.096 mmol/kg), BBA (1.25 mmol/kg), BBA/CM-β-CD (0.096 mmol/kg), BBA/FA-PEG-CM-β-CD (0.096 mmol/kg) with saline as negative control and 5-FU as a positive control. All animals were given a single oral gavage of 0.2 mL of different dosage forms, and their body weight was monitored every day. Meanwhile, the toxicity and mortality of all animals were observed every day. The conventional changes such as the cases of the diet, urine, and feces were recorded. On the 15th day after treatment, the blood of each group was collected from the heart and centrifuged at 1,000 × g for 10 min to collect serum. An automated biochemical analyzer was used to detect serum alanine aminotransferase (ALT), aspartate aminotransferase (AST), blood urea nitrogen (BUN), and creatinine (Cr). Meanwhile, the heart, liver, lung, and kidney were quickly excised, washed with normal saline, and weighed. The visceral index is estimated according to the following formula as Viscera Index (%) = Viscera Weight / Body Weight × 100. And then, all the tissues were fixed with 10% formalin and stained with hematoxylin & eosin (H&E) for pathological analysis.

### Pharmacokinetics evaluation of BBA/FA-PEG-CM-β-CD

SW620 tumor-bearing mice were randomly divided into 3 groups (n = 5). BBA, BBA/CM-β-CD, and BBA/FA-PEG-CM-β-CD were administered by gavage at a dose of 0.366 g/kg. The blood was collected respectively from the heart at 0.25, 0.5, 1, 2, 3, 4, 6, 8, 12, 24, 36, 48, and 72 h after administration. The whole blood was quickly centrifuged at 2000 × g for 5 min, and the upper plasma was precisely aspirated. Then 0.35 mL of methanol was added, vortexed for 2 min, and centrifuged at 3350 × g for 15 min. The supernatant was used to detect butyric acid by HPLC [45]. The pharmacokinetic parameters were calculated by non-compartmental analysis using Drug Analysis System (DAS 2.1.1, Shanghai, China).

### Distribution of BBA/FA-PEG-CM-β-CD in SW620 tumor-bearing mice

The nude mice bearing SW620 xenografts were treated as same as that in the pharmacokinetics experiments. The animals were sacrificed at 1, 3, 8, and 12 h after oral administration. Samples of blood, heart, spleen, liver, kidneys, lung, and tumor tissues were collected quickly (n = 5) to detect butyric acid using the HPLC method.

Meanwhile, the in vivo small animal imaging system (Fx Pro/FX Bruker, USA) was used to investigate the in vivo distribution and targeting ability of BBA/FA-PEG-CM-β-CD. The hydrophobic infrared fluorescent dye DID was encapsulated into the inclusion compound (0.8 μg DID per mice) to form DID-BBA/CM-β-CD and DID-BBA/FA-PEG-CM-β-CD. The nude mice bearing SW620 xenografts were randomized into four groups (n = 5) and given respectively DID-BBA/CM-β-CD and DID-BBA/FA-PEG-CM-β-CD with saline and free DID as controls. Based on the previous work, the animals were anesthetized at 3, 8, and 12 h, and visualized at an excitation wavelength of 644 nm and an emission wavelength of 665 nm. Images of each mouse were normalized to the same intensity range of lowest and highest values, and Carestream MI (Gel Logic 6000 PRO, USA) was used to quantify the fluorescence intensity.

After the in vivo small animal imaging detection, blood was immediately taken from the heart and the nude mice were sacrificed. The whole blood was centrifuged at 1500 × g for 5 min and placed in a 2 mL EP tube. The heart, liver, spleen, kidney, lung, and tumors of nude mice in each group were quickly separated, and the fluorescence intensity of the blood, organs, and tumor tissues was analyzed with the IVIS imaging system [46].

### Pharmacodynamic evaluation of BBA/FA-PEG-CM-β-CD

Ten days after tumor transplantation, the SW620 tumor-bearing mice were randomly divided into 8 groups with 5 mice in each group when the tumors reached about 100 mm^3^. The animals were weighed before treatment, and the measurement was continued every 2 days. The two axes of the tumor were measured with a vernier micrometer (L, longest axis; W, shortest axis). Tumor volume (mm^3^) was calculated as ½ (L × W^2^). Anti-tumor activity was evaluated by tumor growth inhibition (TGI), which was the mean tumor weight (MTW) of the treated group (TG) relative to the saline-treated control group (CG) on day 15, as calculated according to the formula [47]. TGI (%) = (MTW_TG_ − MTW_CG_) / MTW_CG_ × 100.

15 days post-administration, all nude mice were sacrificed. The tumor tissues were collected, stained with H&E, and observed under an inverted microscope (400 ×). In addition, the TUNEL test was performed to detect cell apoptosis [48]. Meanwhile, the expressions of both VEGFR-3 and Ki-67 were detected by ELISA.

### Data analysis

The results were stated as mean ± standard deviation (SD) and analyzed utilizing GraphPad Prism 6.01 (GraphPad Software, La Jolla, CA, USA). One- or two-way analysis of variance was used to statistically compare various groups. The p-value of < 0.05 was considered as a significant difference between groups. The higher significance level was set at P < 0.01.

## Results

### Successful synthesis of BBA and FA-PEG-CM-β-CD

Additional file 1: Figure S1 showed the ^1^H-NMR and ^13^C-NMR spectra of 2-hydroxy-5-butylamino benzoic acid that confirmed the successful synthesis of it. Characteristic peaks of 2-hydroxy-5-butylamino benzoic acid appeared at (g) and (h). Its NMR data were: ^1^H NMR (400 MHz, DMSO): δ 10.97 (s, 1H), 9.84 (s, 1H), 8.10 (s, 1H), 7.64 (s, 1H), 6.88 (s, 1H), 2.24 (t, J = 7.3 Hz, 2H), 1.58 (s, 2H), 0.90 (s, 3H). ^13^C-NMR (101 MHz, DMSO): δ 172.44 (s), 171.39 (s), 157.32 (s), 131.54 (s), 127.82 (s), 120.60 (s), 117.42 (s), 112.93 (s), 39.00 (s), 19.05 (s), 14.25 (s).

Additional file 1: Figure S2 confirmed the successful synthesis of BBA. Characteristic peaks of BBA appeared at (i). Its NMR data were: ^1^H NMR (400 MHz, Acetone) δ 9.29 (s, 1H), 8.29 (s, 1H), 7.96 (s, 1H), 7.08 (s, 1H), 2.54 (s, 2H), 2.36 (s, 2H), 1.70 (s, 4H), 0.98 (d, J = 17.7 Hz, 6H). ^13^C NMR (101 MHz, Acetone) δ 172.79 (s), 172.48 (s), 166.18 (s), 147.33 (s), 138.60 (s), 125.35 (s), 125.12 (s), 123.10 (s), 39.90 (s), 36.73 (s), 30.30 (s), 19.90 (s), 19.11 (s), 14.35 (d, J = 8.6 Hz).

Additional file 1: Figure S3A–C showed the ^1^H NMR spectra of CM-β-CD (A), FA-PEG-NH_2_ (B), and FA-PEG-CM-β-CD (C). The NMR data of FA-PEG-CM-β-CD were: ^1^H NMR (400 MHz, D_2_O) δ 8.67 (s, 1H), 8.28 (m, 1H), 7.59 (s, 1H), 6.69 (s, 1H), 5.24 (s, 10H), 4.23 (s, 8H), 4.03 (s, 2H), 3.79 (s, 31H), 2.79 (d, J = 5.2 Hz, 8H), 1.99 (s, 1H), 1.81 (s, 1H), 1.28 (m, 1H), 1.09 (s, 1H), 0.98 (s, 3H).

The infrared spectrum was shown in Additional file 1: Figure S3D. The characteristic absorption peaks around 3434 cm^−1^ and 1653 cm^−1^ responded to N–H and C = O of the amide bonds in FA-PEG-CM-β-CD (a). The characteristic absorption peak of the conjugated N–H around 3499 cm^−1^ overlapped with the hydroxyl group (O–H) in the spectra of FA-PEG-NH_2_ (c). Peaks due to vibrations of the conjugated O–H bond and C = O bond was detected around 3416 cm^−1^ and 1599 cm^−1^ in the spectra of CM-β-CD (d). These results further proved that FA-PEG-CM-β-CD was successfully synthesized.

### Characterization of BBA/FA-PEG-CM-β-CD

**Fig. 2:**
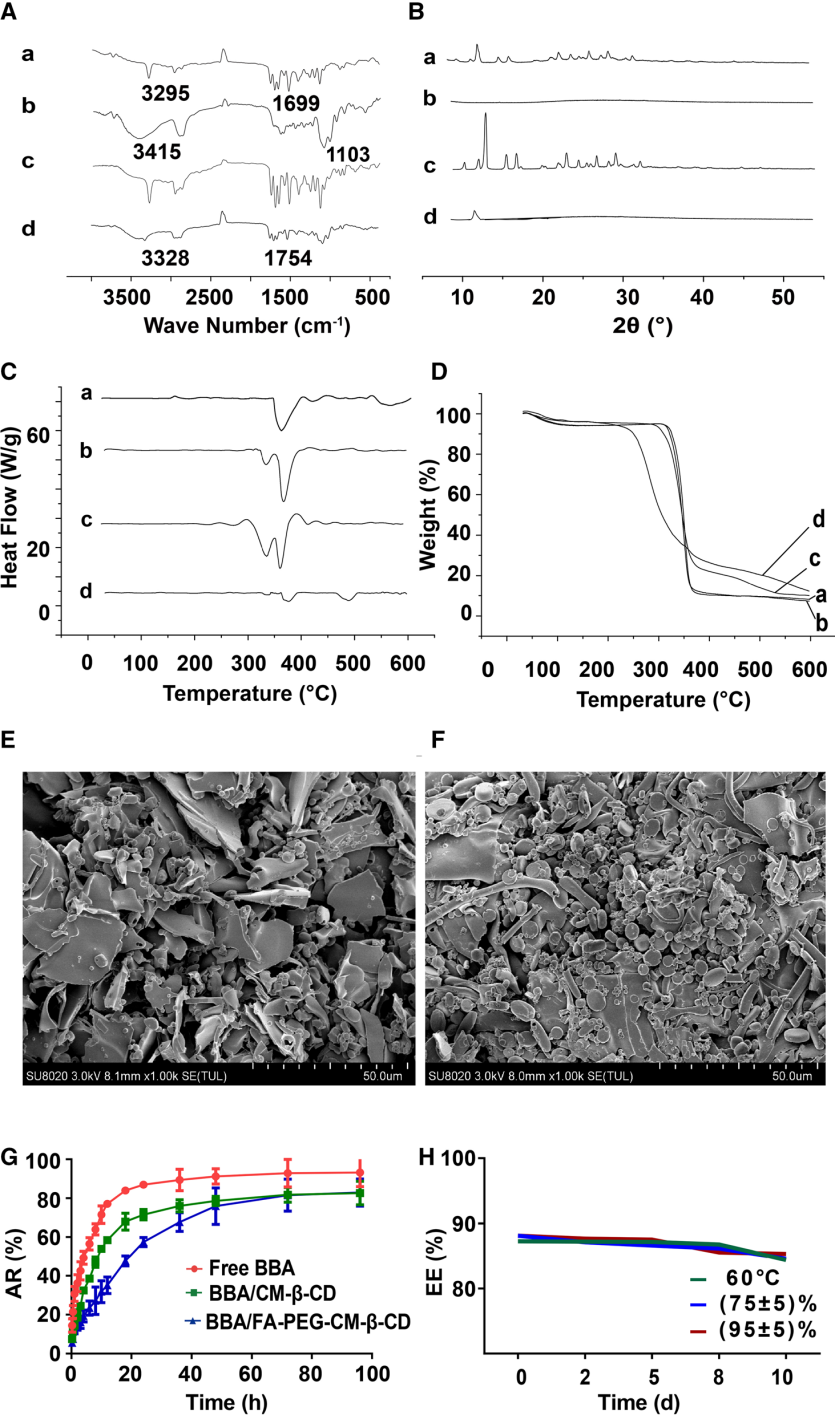
In vitro characterization of BBA/FA-PEG-CM-β-CD. **A** Infrared spectrum, **B** XRD spectrum, **C** Differential scanning calorimetry (DSC) and **D** Thermal gravimetric analysis (TGA) of (a) BBA, (b) FA-PEG-CM-β-CD, (c) mixture of BBA with FA-PEG-CM-β-CD, and (d) BBA/FA-PEG-CM-β-CD. Scanning electron microscopy (SEM) images of **E** FA-PEG-CM-β-CD and **F** BBA/FA-PEG-CM-β-CD were obtained by scanning electron microscopy. **G** Drug release behaviors of BBA, BBA/CM-β-CD, and BBA/FA-PEG-CM-β-CD were assessed in PBS medium (pH7.4) at 37 °C. **H** Changing of entrapment efficiency of BBA/FA-PEG-CM-β-CD under high temperature and high humidity conditions

The infrared spectra were shown in Fig. 2A. A characteristic peak of the conjugated O–H bond was detected near 3295 cm^−1^ in the spectra of BBA (a), and a strong absorption peak of benzene ranged near 1699 cm^−1^. The absorption peaks of BBA and FA-PEG-CM-β-CD (b) did not simply overlap in the infrared spectrum of the BBA/FA-PEG-CM-β-CD (d). Some characteristic peaks of BBA disappeared or weakened, in which the N–H stretching vibration peak near 3295 cm^−1^ disappeared, and a peak due to vibrations of the conjugated C–O–C bond around 1103 cm^−1^ was weakened in the spectra of FA-PEG-CM-β-CD (b). It was concluded that BBA and FA-PEG-CM-β-CD successfully formed the inclusion compound of BBA/FA-PEG-CM-β-CD.

The XRD pattern was shown in Fig. 2B. In the X-ray pattern, the obvious crystal diffraction peak of BBA (a) could be observed, while no obvious crystal diffraction peaks were seen in the FA-PEG-CM-β-CD (b). Compared with the diffraction pattern of BBA (a), the peak position of the BBA/FA-PEG-CM-β-CD inclusion compound (d) shifted, the measurement angle changed, and the characteristic peak disappeared, which preliminarily proved the formation of BBA/FA-PEG-CM-β-CD.

DSC results of BBA (a), FA-PEG-CM-β-CD (b), the mixture of BBA with FA-PEG-CM-β-CD (c), and BBA/FA-PEG-CM-β-CD (d) were shown in Fig. 2C. Two different peaks were observed in BBA (a) and FA-PEG-CM-β-CD (b). However, after BBA was incorporated into FA-PEG-CM-β-CD, the peak at 360 °C decreased greatly. It might be attributed to the formation of BBA/FA-PEG-CM-β-CD.

As shown in Fig. 2D, BBA (a) and FA-PEG-CM-β-CD (b) had a two-step weight loss following as one was below 90 °C due to the water loss, another was ranging 270 °C—390 °C which was the main thermal degradation of BBA and FA-PEG-CM-β-CD. BBA/FA-PEG-CM-β-CD (d) exhibited a 3-step weight loss. The first reduction was seen below 90 °C, due to the water loss of BBA or FA-PEG-CM-β-CD. The other two steps were observed at around 200 °C − 400 °C and 400 °C − 600 °C, which might be attributed to the double thermal degradations of BBA/FA-PEG-CM-β-CD.

As shown in Fig. 2E&F, SEM was used to observe the external morphology of FA-PEG-CM-β-CD (E) and BBA/FA-PEG-CM-β-CD (F). FA-PEG-CM-β-CD existed as lumps. But in the morphological image of BBA/FA-PEG-CM-β-CD, the crystal form was dislocated and the boundary deformed under the action of mechanical force and thus forming small spherical particles that were stacked in some blocks.

The in vitro accumulative release (%) of BBA/FA-PEG-CM-β-CD was investigated by dialysis. As shown in Fig. 2G, BBA/CM-β-CD and BBA/FA-PEG-CM-β-CD released only 82% of BBA in vitro during 96 h, whereas 90% of free BBA was released during the first 24 h. The sustained drug release from BBA/CM-β-CD and BBA/FA-PEG-CM-β-CD with no initial burst release suggested that BBA was not adsorbed on the surface but encapsulated completely inside FA-PEG-CM-β-CD.

Through detecting the encapsulation efficiency of BBA/FA-PEG-CM-β-CD under high temperature and high humidity conditions, it could be seen (Fig. 2H) that as the temperature or humidity increased, the encapsulation rate didn’t change, indicating that BBA/FA-PEG-CM-β-CD in high temperature or high humidity conditions still kept stable.

### In vitro anticancer activity of BBA/FA-PEG-CM-β-CD

**Fig. 3:**
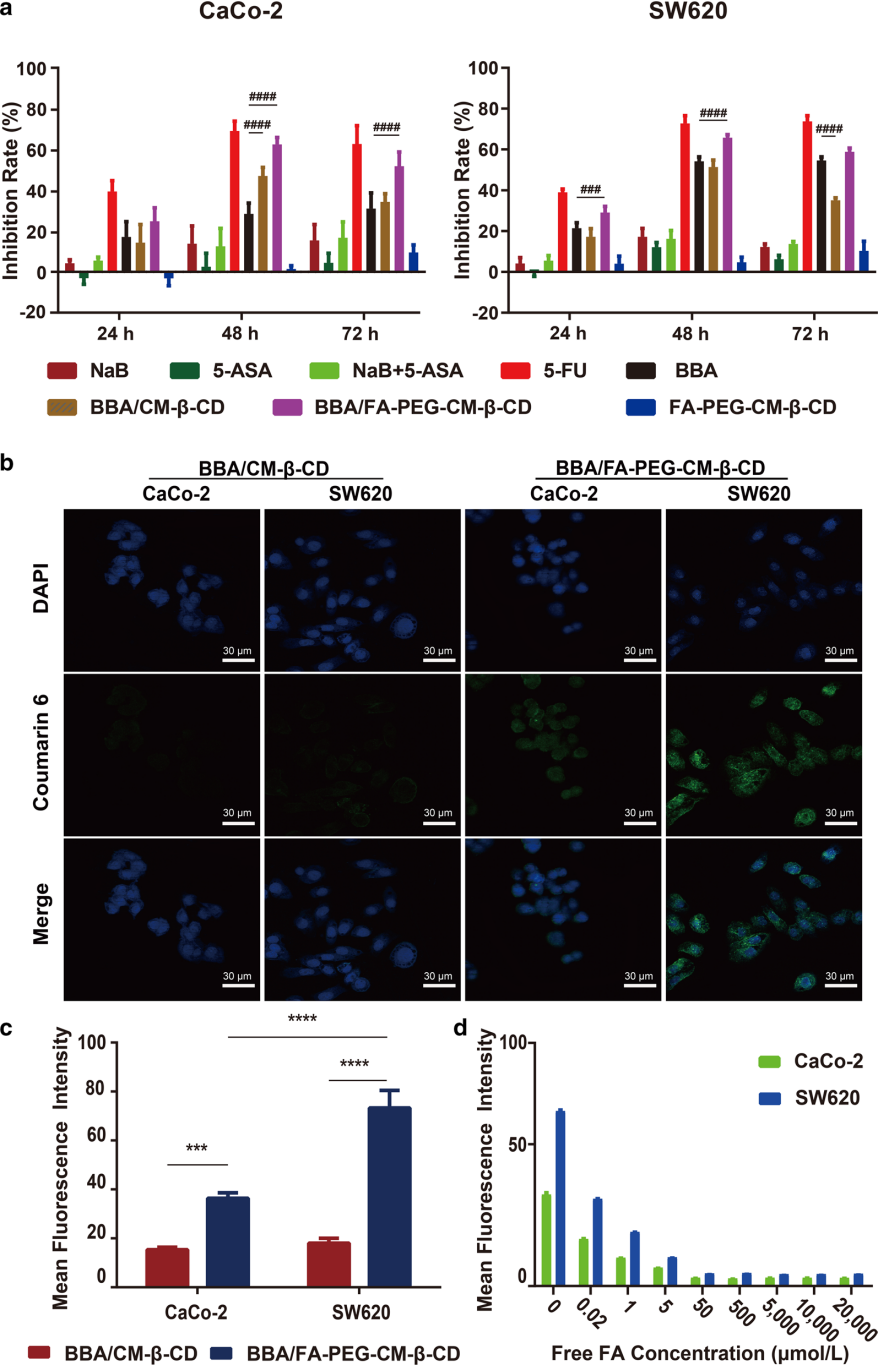
The in vitro cell-growth inhibition effect and cellular uptake of BBA/FA-PEG-CM-β-CD. **A** Cell growth inhibition rate against CaCo-2 and SW620 cells after incubation with different formulations at 30 μM for 48 h, taking 5-FU as the positive control. Cellular uptake detection through **B** confocal laser scanning and **C** the quantitative analysis on the CaCo-2 and SW620 cells. **D** Competitive inhibition on the cell uptake of BBA/FA-PEG-CM-β-CD by the different concentrations of free folic acid (FA). The mean fluorescence intensity was obtained from the green fluorescence intensity through blue fluorescence correction and analyzed by software Image J. The significance of the differences was evaluated using 2-way ANOVA (*P < 0.05, **P < 0.01, ***P < 0.001, ****P < 0.0001). Data were shown as the mean ± SD in each group (n = 3). Symbols^#^ represented the significant difference between the marked group and the BBA group at the same time (^#^P < 0.05, ^##^P < 0.01, ^###^P < 0.001, ^####^P < 0.0001)

MTT assay was used to investigate the cell-growth inhibition effects of BBA/FA-PEG-CM-β-CD at various concentrations against CaCo-2 and SW620 cells at 24 h, 48 h, and 72 h post-treatment (Fig. 3A).

Compared with NaB, the dual-prodrug BBA showed some cell-proliferation inhibition effect on both CaCo-2 and SW620 at the time points of 24 h, 48 h, and 72 h (P < 0.0001). Meanwhile, as prediction the treatment of 5-FU (20 μg/mL) drastically enhanced the cell proliferation inhibition rate compared with the BBA group ( P < 0.0001). BBA/FA-PEG-CM-β-CD showed much better inhibitory effects than BBA, BBA/CM-β-CD. The proliferation inhibition effect of BBA/FA-PEG-CM-β-CD on SW620 cells was better than that on CaCo-2 cells, so we took SW620 cells as the cell model for subsequent tumor-relative evaluation.

### Uptake of BBA/FA-PEG-CM-β-CD by cells in vitro

As shown in Fig. 3B&C, cells treated with BBA/CM-β-CD (30 μM) or BBA/FA-PEG-CM-β-CD (30 μM) showed co-localization of green fluorescence from coumarin 6 and blue fluorescence from DAPI, suggesting that the inclusion complexes had been ingested by the cells. After treatment with BBA/FA-PEG-CM-β-CD, SW620 cells showed much stronger fluorescence signals than CaCo-2, which may be due to that SW620 cells were more sensitive to BBA/FA-PEG-CM-β-CD than CaCo-2 cells. Meanwhile, in BBA/FA-PEG-CM-β-CD group the fluorescence intensity was much greater than that in BBA/CM-β-CD group, demonstrating a targeting ability of BBA/FA-PEG-CM-β-CD mediated by FA ligand.

As shown in Fig. 3D, both CaCo-2 cells and SW620 cells were treated with BBA/FA-PEG-CM-β-CD containing increasing concentration of FA. The uptake of BBA/FA-PEG-CM-β-CD was competitively inhibited by excess FA. This also proved the activity targeting ability of BBA/FA-PEG-CM-β-CD.

### Mechanism analysis about the inhibitive effect of BBA/FA-PEG-CM-β-CD

**Fig. 4:**
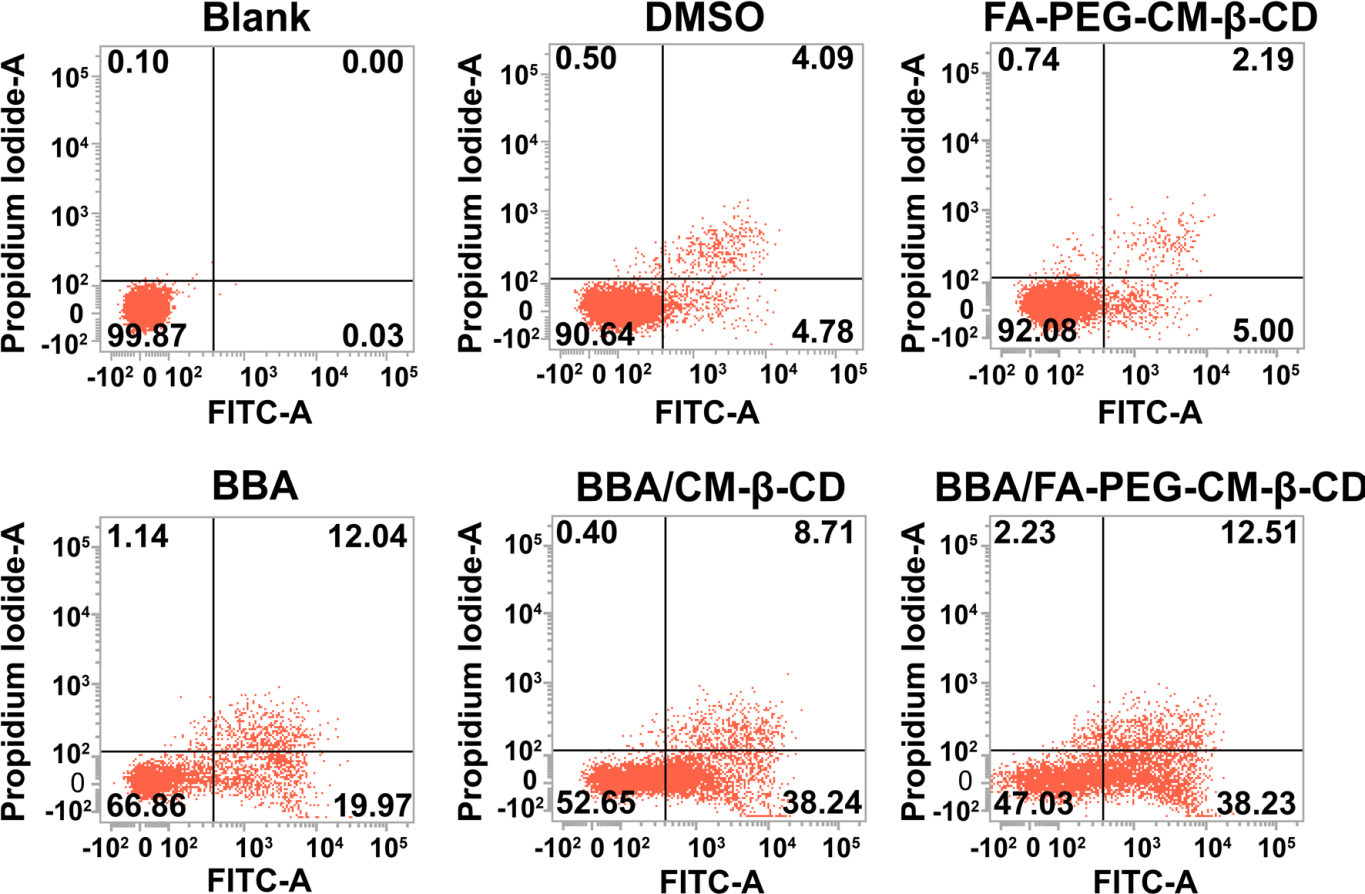
Annexin V/PI binding assay by FACS Scatter plot showing percentage of cells in early apoptotic, late apoptotic, and necrotic quadrants

To fully understand the inhibitive effect mechanism of BBA/FA-PEG-CM-β-CD on the SW620 cells, we further performed cell apoptosis and cell cycle assays. As shown in Fig. 4, SW620 cells were treated with different formulations for 48 h. BBA, BBA/CM-β-CD, and BBA/FA-PEG-CM-β-CD obviously induced apoptosis in SW620 cells compared with the blank group. The apoptotic rates including early apoptosis and late apoptosis were respectively 32.21% ± 8.26%, 46.28% ± 9.4%, and 51.56% ± 8.54% in groups of BBA, BBA/CM-β-CD, and BBA/FA-PEG-CM-β-CD, indicating that BBA/FA-PEG-CM-β-CD induced the strongest apoptosis against SW620 cells.

**Fig. 5:**
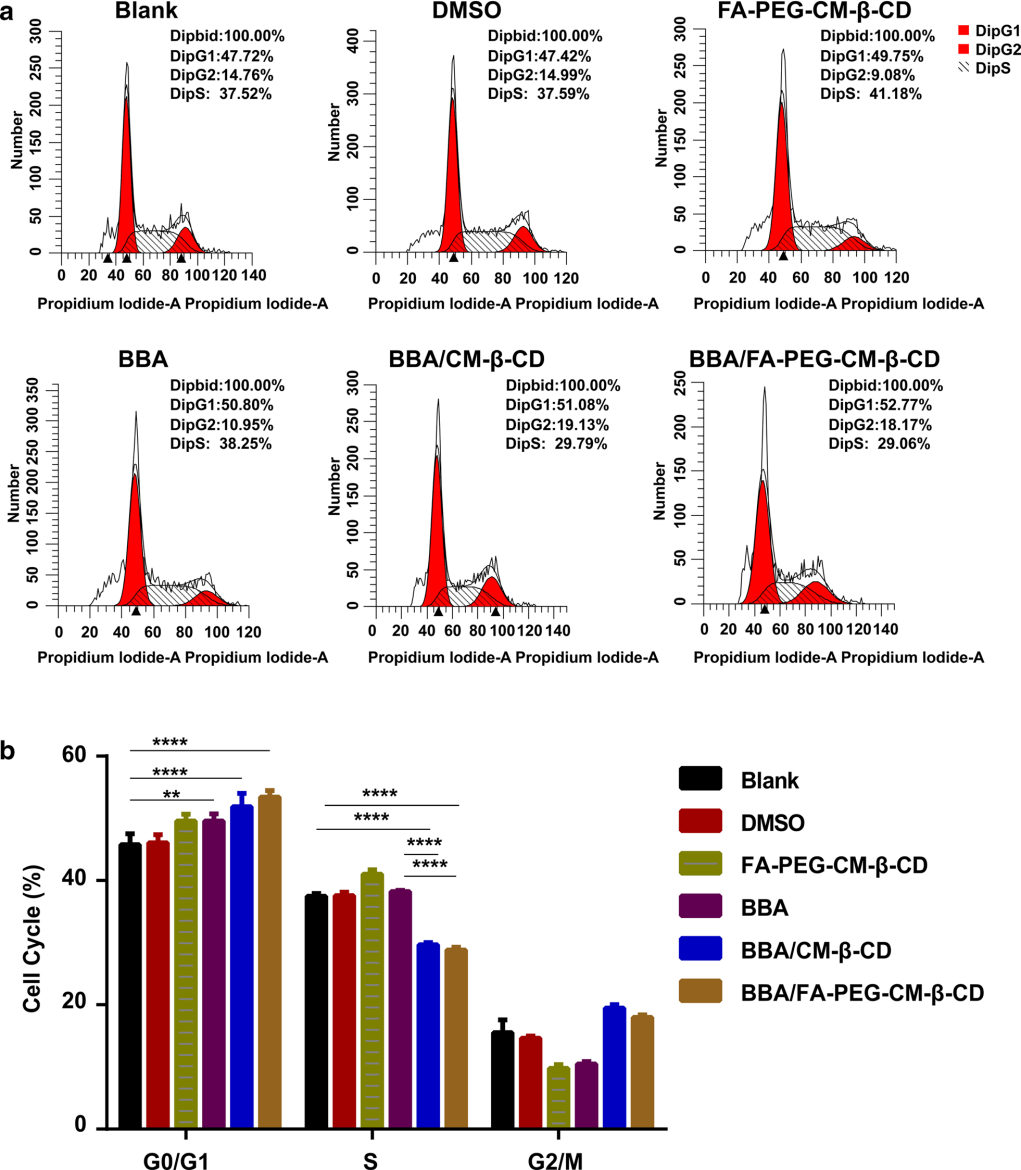
Analysis of cell cycle in SW620 cells. After incubation with different formulation for 48 h, the cells were stained with propidium iodide and detected by flow cytometry (**A**) and quantitative analysis (**B**). *P < 0.05, **P < 0.01, ***P < 0.001, ***P < 0.0001

As shown in Fig. 5, the treatments of BBA, BBA/CM-β-CD, and BBA/FA-PEG-CM-β-CD on SW620 cells induced a significant decrease of cells in the S phase (P < 0.0001) and a significant increase of cells in the G0/G1 phase (P < 0.01), which proved that the SW620 cell was mainly arrested at the G0/G1 phase. Both BBA/CM-β-CD and BBA/FA-PEG-CM-β-CD induced higher cell cycle arrest in G0/G1 phase than BBA.

### BBA/FA-PEG-CM-β-CD showing no in vivo acute toxicity

We examined the in vivo toxicity of BBA/FA-PEG-CM-β-CD in normal Kunming mice. It was found that the hair color, diet, and activities of all mice remained normal. No abnormal behavior, toxic symptoms, bodyweight loss, and death were observed after all the treatments except the 5-FU group. As shown in Table 1, no significant difference in the average body weight was observed among the treatment groups except 5-FU, in which the tested mice treated with 5-FU significantly decreased by 4.46% (P < 0.05). These results demonstrated that BBA/FA-PEG-CM-β-CD showed no toxicity to normal mice.

**Table 1:** The survival and body weight changes of mice in the acute toxicity test

Groups	Amount (start/end)	Bodyweight (g)		Average weight change rate (%)
		Start	End	
Saline	5/5	18.36 ± 1.83	19.64 ± 1.60	6.97
FA-PEG-CM-β-CD	5/5	18.60 ± 0.51	19.28 ± 1.46	3.66
5-ASA	5/5	18.58 ± 0.41	19.44 ± 0.45	4.63
NaB	5/5	18.24 ± 0.53	19.14 ± 0.52	4.93
BBA	5/5	18.48 ± 0.63	18.16 ± 0.63	1.00
5-FU	5/5	18.82 ± 0.61	17.98 ± 0.80**	− 4.46*
BBA/CM-β-CD	5/5	18.57 ± 0.71	19.05 ± 1.59	2.56
BBA/FA-PEG-CM-β-CD	5/5	18.66 ± 0.59	19.74 ± 1.26	5.79

Results were presented as mean ± SD (n = 5). Symbols represented statistical significance compared with saline group. ^⁎^P < 0.05, **P < 0.01

As shown in Table 2, the viscera indexes of heart, liver, lung, and kidney exhibited no significant changes after treatment with BBA and BBA/FA-PEG-CM-β-CD compared with the saline group. However, the liver index increased significantly to 8.34% ± 0.43% after 5-FU treatment. Moreover, the kidney index also increased significantly to 3.06% ± 0.27% after 5-FU treatment, which was significantly different from the saline group (2.30% ± 0.34%, P < 0.01). Compared with 5-FU, the viscera indexes of the mice treated with BBA/FA-PEG-CM-β-CD showed no toxic effect.

**Table 2:** The viscera index of mice treated with different formulations

Groups	Heart index (%)	Liver index (%)	Lung index (%)	Kidney index (%)
Saline	0.79 ± 0.07	7.47 ± 1.36	0.80 ± 0.13	2.30 ± 0.34
FA-PEG-CM-β-CD	0.78 ± 0.06	7.57 ± 1.12	0.82 ± 0.26	2.30 ± 0.36
5-ASA	0.80 ± 0.11	7.49 ± 0.88	0.83 ± 0.16	2.35 ± 0.33
NaB	0.83 ± 0.12	7.61 ± 1.33	0.84 ± 0.31	2.31 ± 0.26
BBA	0.87 ± 0.08	7.05 ± 0.58	0.80 ± 0.21	2.55 ± 0.62
5-FU	0.81 ± 0.28	8.34 ± 0.43*	0.85 ± 0.11	3.06 ± 0.27**
BBA/CM-β-CD	0.80 ± 0.26	7.17 ± 0.63	0.79 ± 0.25	2.17 ± 0.37
FA-PEG-CM-β-CD	0.78 ± 0.06	7.57 ± 1.11	0.82 ± 0.26	2.30 ± 0.36
BBA/FA-PEG-CM-β-CD	0.80 ± 0.11	7.29 ± 0.71	0.81 ± 0.23	2.36 ± 0.24

Results were presented as mean ± S D (n = 5). Symbols represented statistical significance compared with Saline group. *P < 0.05, **P < 0.01

In addition to the viscera index, we further conducted serum biochemistry analyses of ALT, AST, BUN, and Cr to assess the potential toxicities of BBA/FA-PEG-CM-β-CD to the liver and kidney of mice. As shown in Table 3, except for the 5-FU treatment, the serum biochemical analysis indexes of other groups after treatment were all within the normal range, and no significant difference was observed. After 5-FU treatment, AST and ALT increased significantly compared to the saline group (P < 0.05). For the kidney function markers of BUN and Cr, all data were normal in all treatment groups and showed no statistical difference compared to the saline group (P > 0.05), suggesting that all preparations had no obvious toxic effect on the kidney function of the treated mice.

**Table 3:** The blood biochemical parameters of serum from mice treated with different formulations

Groups	ALT (U/L)	BUN (mmol/L)	Cr (μmol/L)	AST (U/L)
Saline	24.2 ± 6.11	8.22 ± 0.97	9.60 ± 3.58	59.5 ± 3.89
FA-PEG-CM-β-CD	19.40 ± 0.82	6.20 ± 1.21	13.57 ± 4.18	59.60 ± 1.01
5-ASA	23.20 ± 2.87	7.82 ± 2.96	9.55 ± 0.36	65.30 ± 1.06
NaB	17.37 ± 1.77	8.60 ± 0.98	8.32 ± 0.98	58.17 ± 4.75
BBA	18.53 ± 0.65	6.32 ± 0.97	8.63 ± 1.96	57.27 ± 10.20
5-FU	292.50 ± 6.60**	6.92 ± 1.20	7.45 ± 2.91	254.00 ± 53.4**
BBA/CM-β-CD	20.77 ± 4.97	6.44 ± 3.60	13.07 ± 1.48	68.17 ± 6.51
BBA/FA-PEG-CM-β-CD	23.87 ± 0.75	7.52 ± 1.57	9.45 ± 1.70	61.87 ± 4.80

Results were presented as mean ± S D (n = 5). Symbols represented statistical significance compared with the saline group

*ALT* alanine aminotransferase, *AST* aspartate aminotransferase, *Cr* creatinine, *BUN* blood urea nitrogen. **P < 0.01

**Fig. 6:**
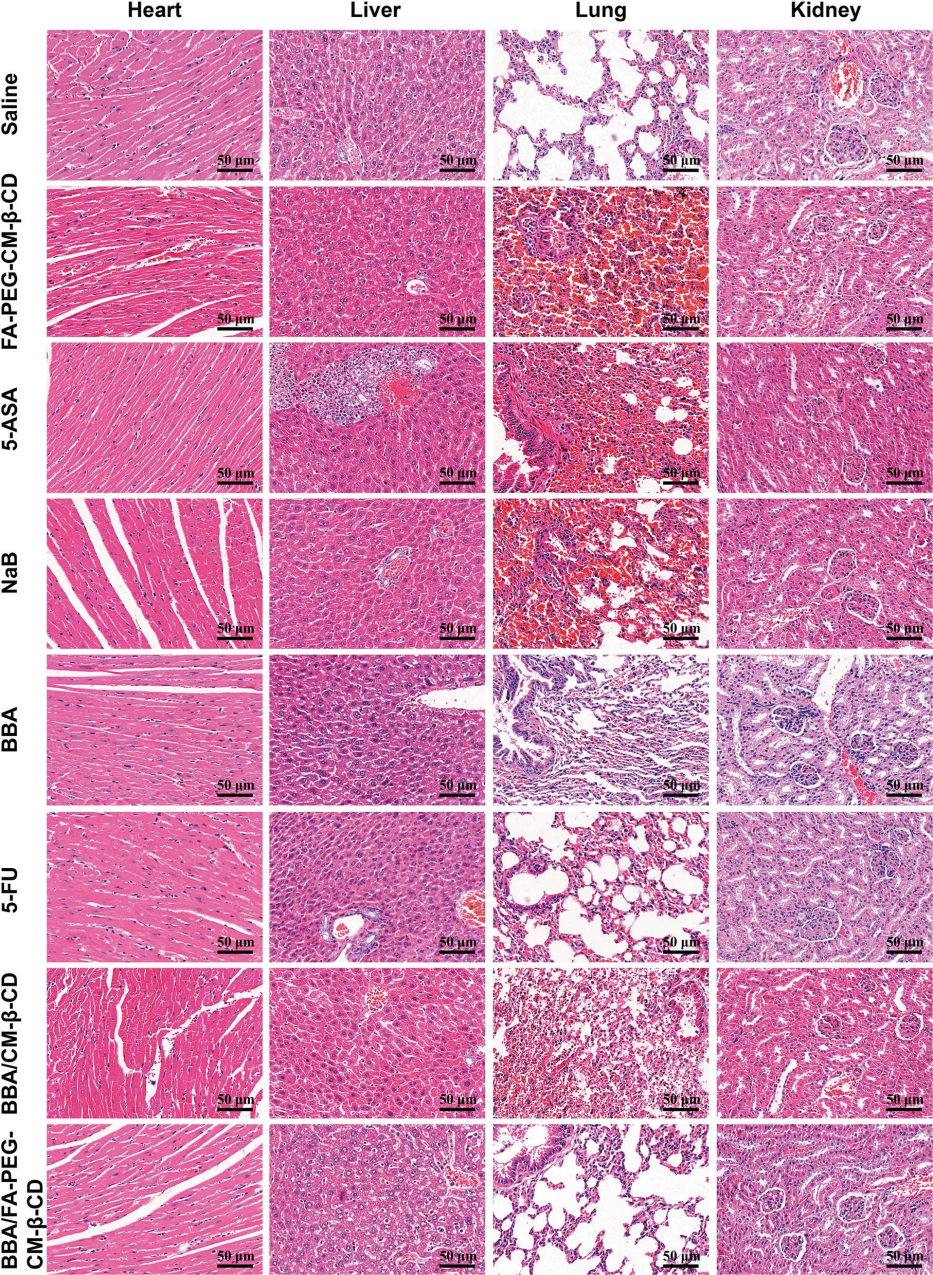
Representative images (×400) of hematoxylin and eosin (H&E) staining of heart, liver, lung, and kidney from mice treated with 200 μL of different solutions as following: saline as control, 5-ASA, NaB, BBA, 5-FU, BBA/CM-β-CD, and BBA/FA-PEG-CM-β-CD

We also performed histological studies of the heart, liver, kidney, and lung after treatment with the different formulation mentioned above. At least three sections for each mouse from each group (n = 5) were randomly and blindly analyzed by a pathologist who was blinded to the experimental protocol. The experimental results were shown in Fig. 6. First, it was found that no obvious histological changes appeared in the heart, liver, kidney, and lung of mice treated with the different formulation. Secondly, the lungs of nude mice treated by each group showed unequal amounts of inflammatory cell infiltration in the interstitium of lung tissues, which might be related to tumor growth. Lastly, different degree of bleeding was observed in the lung from 5-ASA, NaB, FA-PEG-CM-β-CD, BBA/CM-β-CD treatment groups, which might be related to improper operation during dissection. The heart, liver, or lung, and kidney tissues of all treatment groups showed no pathological injury related to the treatment of this test, which further proved the non-toxicity of BBA/FA-PEG-CM-β-CD to normal mice.

### BBA/FA-PEG-CM-β-CD prolonging the in vivo circulation time of BBA

**Fig. 7:**
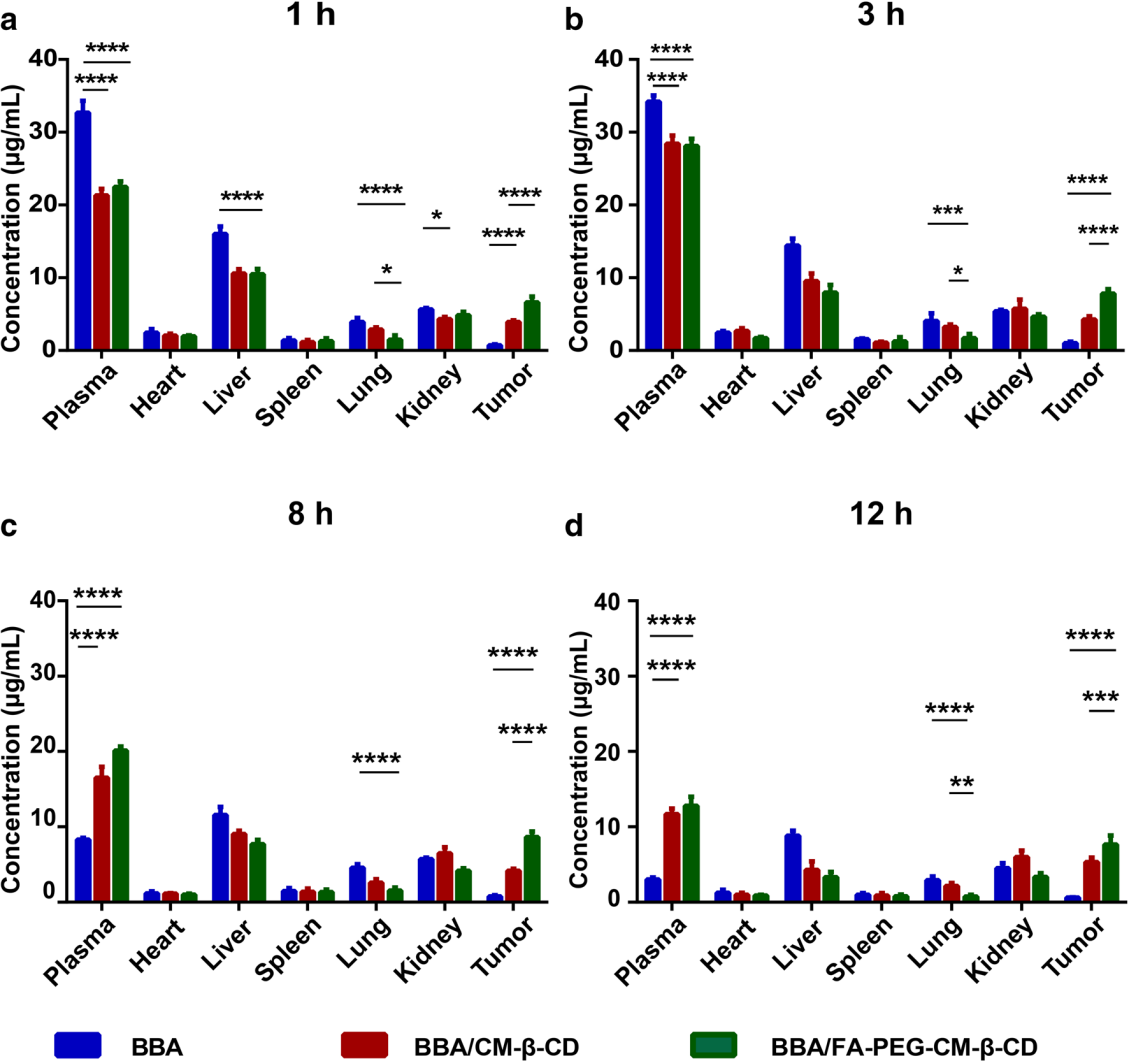
In vivo concentration of butyric acid from BBA, BBA/CM-β-CD and BBA/FA-PEG-CM-β-CD at 3, 8, 12 h in the SW620 tumor-bearing nude mice. The significance of the differences was evaluated using 2-way ANOVA (*P < 0.05, **P < 0.01, ***P < 0.001 and ****P < 0.0001). Data were shown as the mean ± SD in each group (n = 5)

The SW620 tumor-bearing nude mice were given orally with free BBA, BBA/CM-β-CD, and BBA/FA-PEG-CM-β-CD. The blood concentration of butyric acid was determined at different time points. As shown in Fig. 7A, butyric acid showed a rapid release when delivered as free BBA, but sustained release when delivered as BBA/CM-β-CD or BBA/FA-PEG-CM-β-CD, which was consistent with the in vitro release study. The blood-concentration of butyric acid showed no significant difference between BBA/CM-β-CD and BBA/FA-PEG-CM-β-CD, suggesting that the FA-PEG-NH_2_ modification did not affect the metabolism of BBA/CM-β-CD. Pharmacokinetic parameters (Table 4) indicated that loading BBA into FA-PEG-CM-β-CD could significantly prolong the in vivo circulation time of BBA*.*

**Table 4:** Pharmacokinetic parameters of BBA, BBA/CM-β-CD and BBA/FA-PEG-CM-β-CD in plasma of BALB/C nude mice after oral administration (n = 5)

Parameters	Unit	BBA	BBA/CM-β-CD	BBA/FA-PEG-CM-β-CD
C_max_	μg/mL	35.923 ± 0.826	28.545 ± 0.716	29.445 ± 0.536
AUC_0-t_	μg/mL*h	219.263 ± 5.360	348.252 ± 10.121	373.902 ± 4.735
MRT_0-t_	h	4.769 ± 0.115	8.454 ± 0.026	8.850 ± 0.065
T_1/2_	h	3.396 ± 0.791	6.694 ± 1.076	8.617 ± 1.422
CL	mL/h/kg	12.760 ± 0.268	7.337 ± 0.413	6.881 ± 0.699

To assess the targeted ability of BBA/FA-PEG-CM-β-CD to tumor tissue, we analyzed the tissue distribution of BBA, BBA/CM-β-CD, or BBA/FA-PEG-CM-β-CD in the SW620 tumor-bearing nude mice after oral administration. As shown in Fig. 7B, in the first 3 h butyric acid concentration in plasma was lower from both BBA/CM-β-CD and BBA/FA-PEG-CM-β-CD than that from BBA (P < 0.0001). But at time points of 8 h (Fig. 7C) and 12 h (Fig. 7D), the blood concentration of butyric acid from BBA/CM-β-CD and BBA/FA-PEG-CM-β-CD was significantly higher than that from BBA (P < 0.0001), which showed that the drug-carrying inclusion compound made by encapsulating BBA with CM-β-CD could significantly prolong the circulation time of BBA. The butyric acid concentration in the heart, spleen, and lung was kept at low levels in all groups. Both BBA/CM-β-CD and BBA/FA-PEG-CM-β-CD were mainly distributed in blood, liver, kidney, and tumor tissues. In tumor tissue, concentrations of both BBA/CM-β-CD and BBA/FA-PEG-CM-β-CD were higher than that of BBA at all time points, in which BBA/FA-PEG-CM-β-CD accumulated significantly more in tumor than BBA/CM-β-CD.

**Fig. 8:**
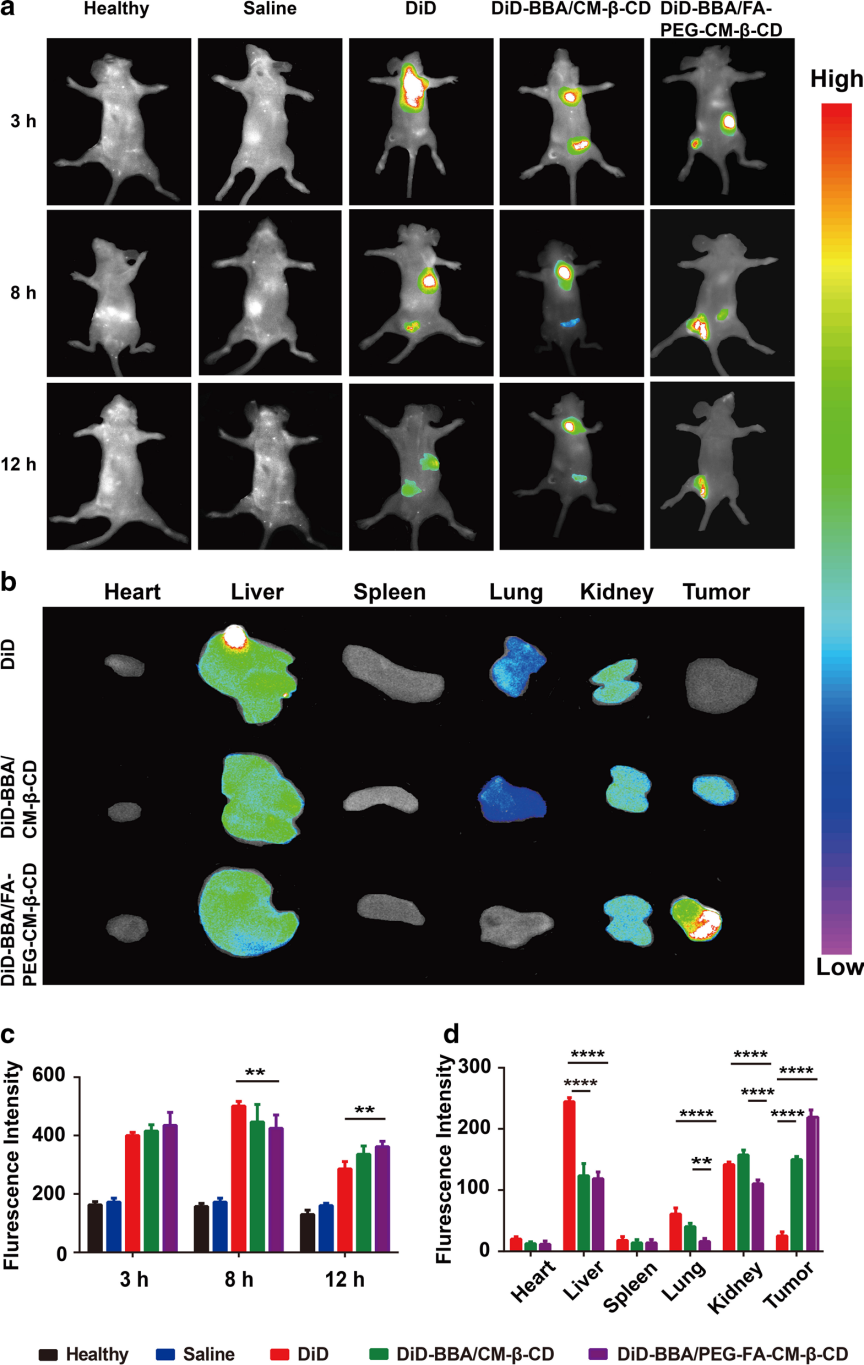
In vivo fluorescence imaging of SW620 tumor-bearing nude mice after administration with DiD, DiD-BBA/CM-β-CD, and DiD-BBA/FA-PEG-CM-β-CD. **A** Fluorescence distribution observed using an in vivo imaging system at 3, 8, and 12 h post-administration. **B** Ex vivo fluorescence imaging of the heart, liver, spleen, lung, kidney, and tumor tissue of SW620 tumor-bearing mice at 12 h post-administration. Semi-quantitative analysis of **C** the in vivo fluorescence intensity at three-time points and **D** ex vivo fluorescence intensity of tissues using ImageJ. Data were expressed as mean ± standard deviation (n = 5). Two-way ANOVA (**P < 0.01, ***P < 0.001, ****P < 0.0001) was used to assess the statistical differences between the treatment groups

Further, the tissue distribution of BBA/FA-PEG-CM-β-CD was detected by in vivo small animal imaging system (Fig. 8). The fluorescence in tumor tissues was significantly stronger from DiD-BBA/FA-PEG-CM-β-CD than that from DiD or DiD-BBA/CM-β-CD (P < 0.0001). All three treatments showed obvious fluorescence in the liver and kidney in the SW620 tumor-bearing mice. But DiD-BBA/FA-PEG-CM-β-CD group could be observed a lower level of fluorescence in the kidney than the DiD-BBA/CM-β-CD group. The fluorescence intensity in the liver was significantly lower from DiD-BBA/CM-β-CD and DiD-BBA/FA-PEG-CM-β-CD than that from DID.

From these results mentioned above, it could be concluded that the FA-modified CM-β-CD inclusion compound could prolong the half-life of BBA, and this novel drug delivery system showed a certain tumor-targeting effect.

### BBA/FA-PEG-CM-β-CD showing tumor suppression to colon cancer

**Fig. 9:**
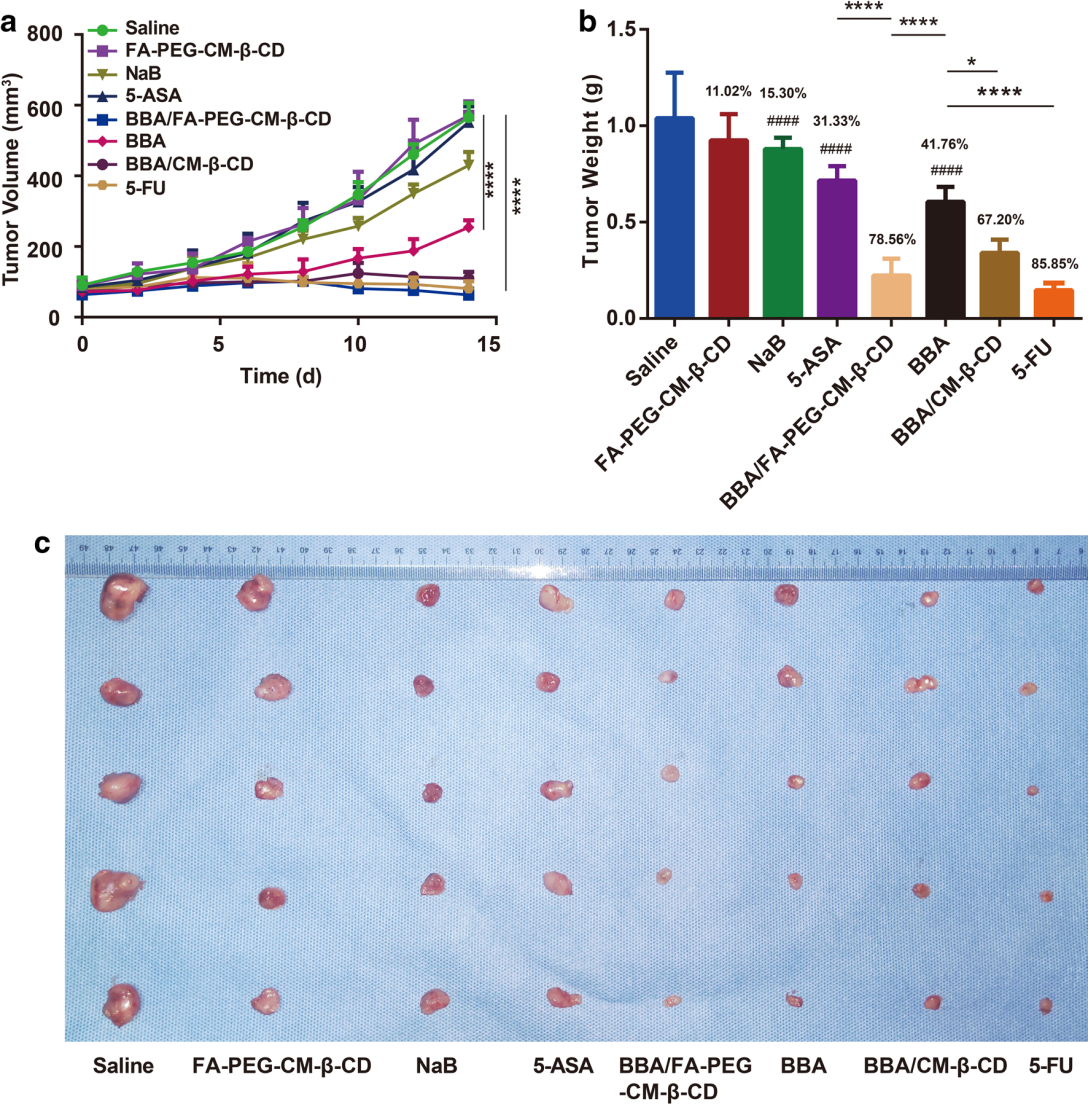
Anti-tumor effect of BBA/FA-PEG-CM-β-CD on SW620 xenograft model. The SW620 tumor-bearing nude mice in each group were initially given the different formulation on 10 days post-inoculation every two days for 7 times as following: saline (as control), FA-PEG-CM-β-CM, NaB, 5-ASA, BBA/FA-PEG-CM-β-CD, BBA, BBA/CM-β-CD, and 5-FU. Tumor volume (mm^3^) was monitored every 2 days after the first administration (**A**). On 25 days post-implantation, the nude mice were euthanized. The tumor weight and tumor-growth inhibition rate (**B**) were measured, and representative images (**C**) of tumor tissue from each group were presented

The SW620 tumor-bearing mice were sacrificed by euthanasia on day 25 after tumor implantation. The results presented in Fig. 9 showed the kinetics of the antitumor activities of the different formulation against SW620 xenografts in nude mice. Figure 9A showed that in the BBA/FA-PEG-CM-β-CD-treated group the mean tumor volume increased very slowly, compared to the saline group (P < 0.0001), indicating that BBA/FA-PEG-CM-β-CD had a significant anti-tumor effect. Meanwhile, BBA/FA-PEG-CM-β-CD gave the anti-tumor effect much higher than BBA.

As shown in Fig. 9B, the mean tumor inhibitory rates in nude mice treated with 5-ASA, NaB, BBA, 5-FU, BBA/CM-β-CD and BBA/FA-PEG-CM-β-CD were 31.33% ± 7.17%, 15.30% ± 5.61%, 41.76% ± 7.56%, 85.85% ± 2.89%, 67.20% ± 6.57%, and 78.56% ± 8.48% against SW620 tumors, respectively. Compared with NaB and 5-ASA, BBA enhanced the tumor inhibition effect, which may result from the combination therapy of anti-inflammatory role of 5-ASA and anti-tumor effect of NaB to colon cancer. Moreover, BBA/FA-PEG-CM-β-CD produced a significant decrease in the tumor weight of mice compared to BBA (P < 0.05), which showed great tumor suppression as well as the positive control of 5-FU. These results demonstrated that the delivery system of BBA/FA-PEG-CM-β-CD had a good inhibitory effect on SW620 xenograft tumors while showing no acute toxicity to mice.

### BBA/FA-PEG-CM-β-CD inducing tumor cells necrosis and apoptosis

**Fig. 10:**
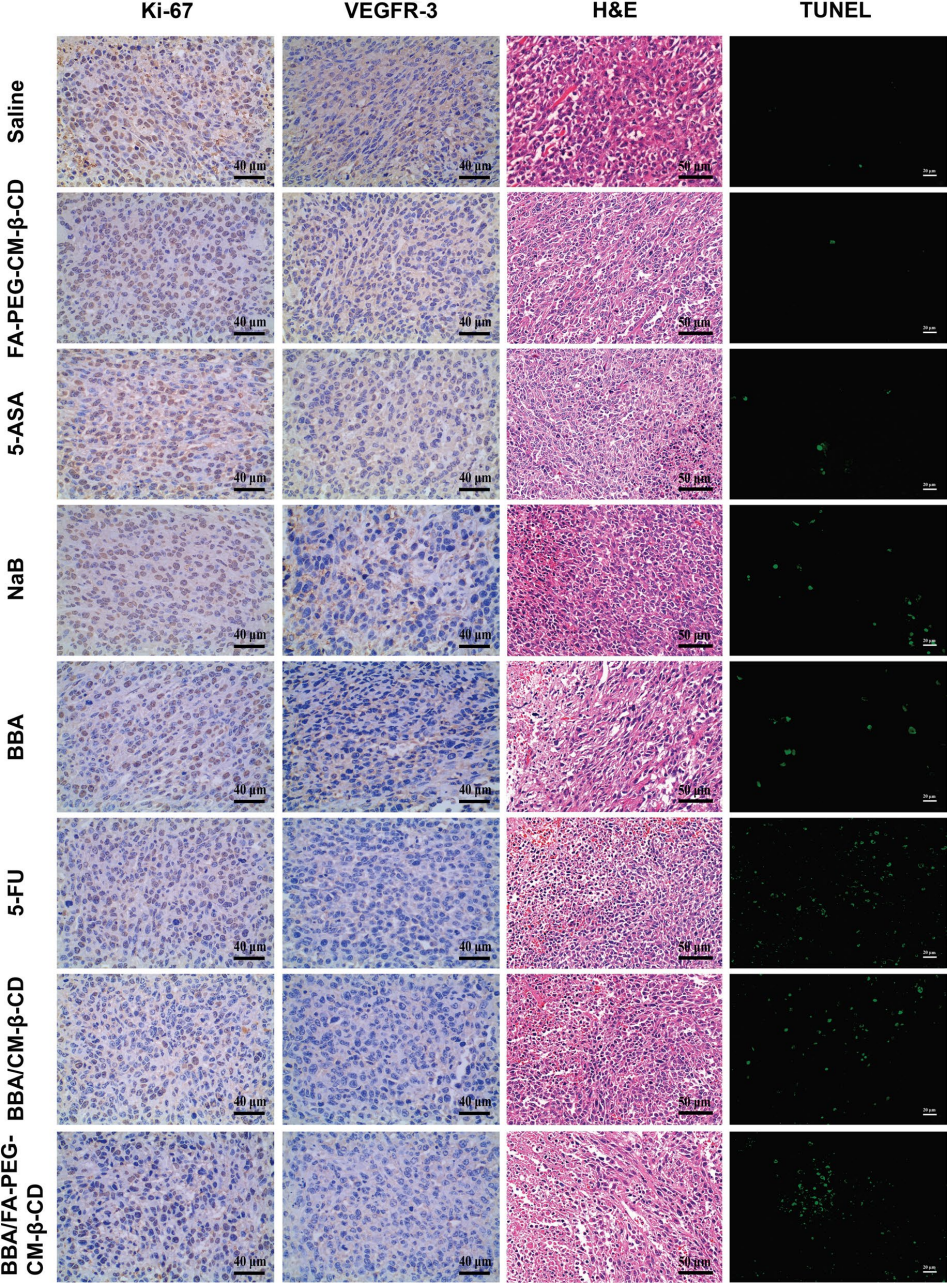
Anti-tumor mechanism assay of BBA/FA-PEG-CM-β-CD (×400). Tumor sections were excised, fixed, dewaxed, and conducted to analyze the endothelial cell marker VEGFR, and the cell proliferation marker ki-67, H&E staining for determining necrosis rate, and TUNEL assay for detection of apoptosis, respectively

To further confirm the antitumor activity of BBA/FA-PEG-CM-β-CD, pathological analysis of tumor tissues was performed by H&E assay. As shown in Fig. 10, some typical necrosis, such as nuclear fragmentation, shrink, and dissolution, was observed in tumor-bearing mice treated with 5-FU, BBA, BBA/CM-β-CD, and BBA/FA-PEG-CM-β-CD. The average necrosis rates were 20%, 50%, 50%, 30%, 10%, and 15% for BBA, BBA/FA-PEG-CM-β-CD, 5-FU, BBA/CM-β-CD, NaB, and 5-ASA, which was consistent with the antitumor effect, suggesting that BBA/FA-PEG-CM-β-CD could cause tumor cell necrosis, leading to tumor growth inhibition.

For evaluation of the apoptosis rate, we chose multiple fields to calculate the average fluorescence intensity in the TUNEL analysis. Results showed that the apoptosis rates from groups of saline, FA-PEG-CM-β-CD, NaB, 5-ASA, BBA/FA-PEG-CM-β-CD, BBA, BBA/ CM-β-CD and 5-FU were 0.06%, 0.75%, 1.45%, 2.44%, 5.13%, 3.27%, 4.49%, and 8.87%, respectively. From these results, it can be seen that a higher level of apoptosis rate was observed in the tumor of mice treated with BBA/FA-PEG-CM-β-CD which showed obvious anti-tumor activity. It is consistent with the results showed in Fig. 9.

To further determine whether the antitumor effect of BBA/FA-PEG-CM-β-CD was associated with the antiangiogenic effect, we analyzed the endothelial cell marker VEGFR-3 and the cell proliferation marker Ki-67. The VEGFR-3 positive area was significantly reduced in animals treated with NaB, 5-ASA, 5-FU, BBA, BBA/CM-β-CD, and BBA/FA-PEG-CM-β-CD, respectively. A higher degree reduction in vascularity was observed in SW620 xenografts after treatment with 5-FU and BBA/FA-PEG-CM-β-CD. In these sections, the reduced VEGFR-3 staining was accompanied by tumor necrosis. In terms of the Ki-67 detection, a significant decrease of the positive expression was observed in mice treated 5-FU and BBA/FA-PEG-CM-β-CD, indicating that the BBA/FA-PEG-CM-β-CD effectively inhibited tumor cell proliferation in vivo as well as 5-FU.

## Discussion

FA-PEG-CM-β-CD was prepared by chemical synthesis method using functional PEG as the linking arm, and then BBA/FA-PEG-CM-β-CD was prepared by a saturated aqueous solution method. At the beginning of the synthesis experiment, we used FA directly to connect CM-β-CD through chemical synthesis. However, it was found that the solubility of FA was too poor to increase yield and the product of FA-CM-β-CD was also difficult to dissolve in water, which was inconsistent with the expectations to enhance the water solubility of the drug. Therefore, we switched to using HOOC-PEG_2000_-NH_2_ as the linker to connect CM-β-CD with FA to form FA-modified cyclodextrin of FA-PEG-CM-β-CD [49], in which PEG could provide multiple advantages such as the prolonged half-life, reduced immunogenicity, higher biological stability, better water solubility, and specific targeting ability to cells or tissues.

BBA/FA-PEG-CM-β-CD was proved to have good targeting ability to the FA-positive SW620 cells in vitro. Meanwhile, BBA/FA-PEG-CM-β-CD could enhance the stability of BBA, protect BBA from being degraded by gastrointestinal metabolism, and significantly improve cellular uptake in SW620 cells. The results from the MTT assay and the in vitro cell apoptosis detection showed that BBA/FA-PEG-CM-β-CD enhanced the cell growth inhibitory effect on SW620 cells, and induced cell apoptosis. In animal pharmacodynamic studies, the difference in tumor suppression in each treatment group also achieved the above-mentioned similar inhibitory effect. Both the in vitro inhibition effects on cellular proliferation and the antitumor efficacy on SW620 tumor-bearing nude mice showed dose-dependent and time-dependent manners.

Due to the superiority of the oral route of administration [50], the prepared cyclodextrin inclusion complexes were given orally rather than intravenously. Then, through the well-known colon targeting material of CM-β-CD, loading BBA with FA-PEG-CM-β-CD to prepare the inclusion complex of BBA/FA-PEG-CM-β-CD could protect BBA from the absorption or degradation in the upper gastrointestinal tract. Under the specific alkaline conditions of the colon site and the action of special enzymes, BBA/FA-PEG-CM-β-CD could be degraded and produce the original butyric acid and 5-ASA. Meanwhile, due to the folate receptor, some intact BBA/FA-PEG-CM-β-CD may directly enter the tumor cells and degrade into butyric acid and 2-hydroxyl-5-butylaminobenzoic at the tumor acidic environment to play the anti-tumor and anti-inflammatory effects.

The in vitro inhibition effects on cellular proliferation showed that the cell proliferation inhibition rate of BBA was significantly higher than that of sodium butyrate and 5-ASA, proving that the anti-cancer effect of BBA was significantly higher than that of NaB and 5-ASA raw materials alone. This result indicated that the dual-prodrug BBA himself had some anti-tumor effects, which may be due to that BBA was degraded by the special environment of tumor cells or that BBA need not revert to the original forms to play the anti-tumor role. The specific mechanism is not yet clear and needs further study. The results from the TUNEL experiment also obtained a similar anti-cancer effect, which may contribute to the combination of inhibiting histone deacetylation by butyric acid, and inhibiting NF-κB and scavenging free radicals by 5-ASA [4, 20]. In addition, the in vitro cell proliferation inhibition rate of 5-FU was significantly higher than that of BBA and BBA/CM-β-CD, but slightly higher than that of the BBA/FA-PEG-CM-β-CD. Compared with CaCo-2 cells, BBA/FA-PEG-CM-β-CD acted on SW620 cells with a higher cell proliferation inhibition rate, indicating that BBA/FA-PEG-CM-β-CD was more sensitive to SW620 cells. Therefore, we chose SW620 cells as the experimental cell model for the cellular and animal experiments. The results of apoptosis detection showed significant differences among the groups of BBA, BBA/CM-β-CD, and BBA/FA-PEG-CM-β-CD, but a similar difference was not observed in that of cell cycle experiment among them, indicating that BBA, BBA/CM-β-CD, and BBA/FA-PEG-CM-β-CD inhibited SW620 cells through the apoptosis mechanism rather than the mechanism of G0/G1 cell cycle arrest [51].

## Conclusion

In the present study, we have developed a novel dual-prodrug BBA by linking butyryl chloride and butyric anhydride to 5-ASA through a two-step chemical reaction. Further, we have developed a novel inclusion complex of BBA/FA-PEG-CM-β-CD modified with FA and revealed that the inclusion complex significantly enhanced the antitumor effect on SW620 carcinoma in vitro and in vivo while showing no toxicity to the tested mice. Although it did not show a higher tumor suppression effect than the widely used 5-FU in clinical application, BBA/FA-PEG-CM-β-CD showed no toxicity to the tested mice, and thus it may have great potential for the clinical treatment of colon cancer.

## Supplementary Information

**Additional file 1: Figure S1.** Structural characterization of 2-hydroxy-5-butylamino benzoic acid. 1H-NMR spectra (A) and 13C-NMR spectra (B) of 2-hydroxy-5-butylamino benzoic acid in DMSO. **Figure S2.** Structural characterization of BBA. 1H-NMR spectra (A) and 13C-NMR spectra (B) of BBA in (CD3)2CO. **Figure S3.** Structural characterization of FA-PEG-CM-β-CD. 1H-NMR spectra of CM-β-CD (A), FA-PEG-NH2 (B), and FA-PEG-CM-β-CD (C). Fourier transform infrared spectra (D) of FA-PEG-CM-β-CD (a), mixture of FA-PEG-NH2 and CM-β-CD (b), FA-PEG-NH2 (c), and CM-β-CD (d). **Figure S4.** Particle size distribution of BBA/FA-PEG-CM-β-CD (DOCX 35712 KB)

## Data Availability

All data generated or analysed during this study are included in this manuscript and its additional file.

## Abbreviations

5-ASA: 5-Aminosalicylic acid
5-FU: 5-Fluorouracil
ALT: Alanine aminotransferase
AST: Aspartate aminotransferase
BA: Butyric acid
BBA: 2-Butyryl oxy-5-butylaminyl benzoic acid
BUN: Blood urea nitrogen
CDs: Cyclodextrins
CG: Control group
CM-β-CD: Carboxymethyl-β-cyclodextrin
Cox-2: Cyclooxygenase-2
Cr: Creatinine
DAS: Drug analysis system
DL: Drug-loading rate
DMEM: Dulbecco’s Modified Eagle’s Medium
DSC: Differential scanning calorimetry
EDC: 1-Ethyl-3-(3-dimethylaminopropyl) carbodiimide hydrochloride
EE: Encapsulation efficiency
FA: Folic acid
FBS: Fetal bovine serum
FR: Folate receptor
FTIR: Fourier transformed infrared
HAT: Histone acetylases
HDAC: Histone deacetylase
HPLC: High-performance liquid chromatography
IL-1β: Interleukin-1β
MTT: Thiazolyl blue tetrazolium bromide
MTW: Mean tumor weight
NaB: Sodium butyrate
NF-κB: Nuclear factor-k-gene binding
NHS: N-hydroxysuccinimide
PEG: Polyethylene glycol
SEM: Scanning electron microscopy
TG: Treated group
TGA: Thermal gravimetric analysis
TG-DSC: Thermal gravimetric-differential scanning calorimetry
TGI: Tumor growth inhibition
TNF-α: Tumor Necrosis Factor-α
XRD: X-ray diffraction
