# Supplementary material for: A novel dual-prodrug carried by cyclodextrin inclusion complex for the targeting treatment of colon cancer
Source: J Nanobiotechnology. 2021 Oct 19;19:329. doi: 10.1186/s12951-021-01064-3 (PMC8524854; doi:10.1186/s12951-021-01064-3)
Supplement: Supplementary file 1 — Additional file 1: Figure S1. Structural characterization of 2-hydroxy-5-butylamino benzoic acid. 1H-NMR spectra (A) and 13C-NMR spectra (B) of 2-hydroxy-5-butylamino benzoic acid in DMSO. Figure S2. Structural characterization of BBA. 1H-NMR spectra (A) and 13C-NMR spectra (B) of BBA in (CD3)2CO. Figure S3. Structural characterization of FA-PEG-CM-β-CD. 1H-NMR spectra of CM-β-CD (A), FA-PEG-NH2 (B), and FA-PEG-CM-β-CD (C). Fourier transform infrared spectra (D) of FA-PEG-CM-β-CD (a), mixture of FA-PEG-NH2 and CM-β-CD (b), FA-PEG-NH2 (c), and CM-β-CD (d). Figure S4. Particle size distribution of BBA/FA-PEG-CM-β-CD (DOCX 35712 KB) [file 12951_2021_1064_MOESM1_ESM.docx]

**Additional file**





Figure S1. Structural characterization of 2-hydroxy-5-butylamino benzoic acid. ^1^H-NMR spectra (A) and ^13^C-NMR spectra (B) of 2-hydroxy-5-butylamino benzoic acid in DMSO.





Figure S2. Structural characterization of BBA. ^1^H-NMR spectra (A) and ^13^C-NMR spectra (B) of BBA in (CD_3_)_2_CO.





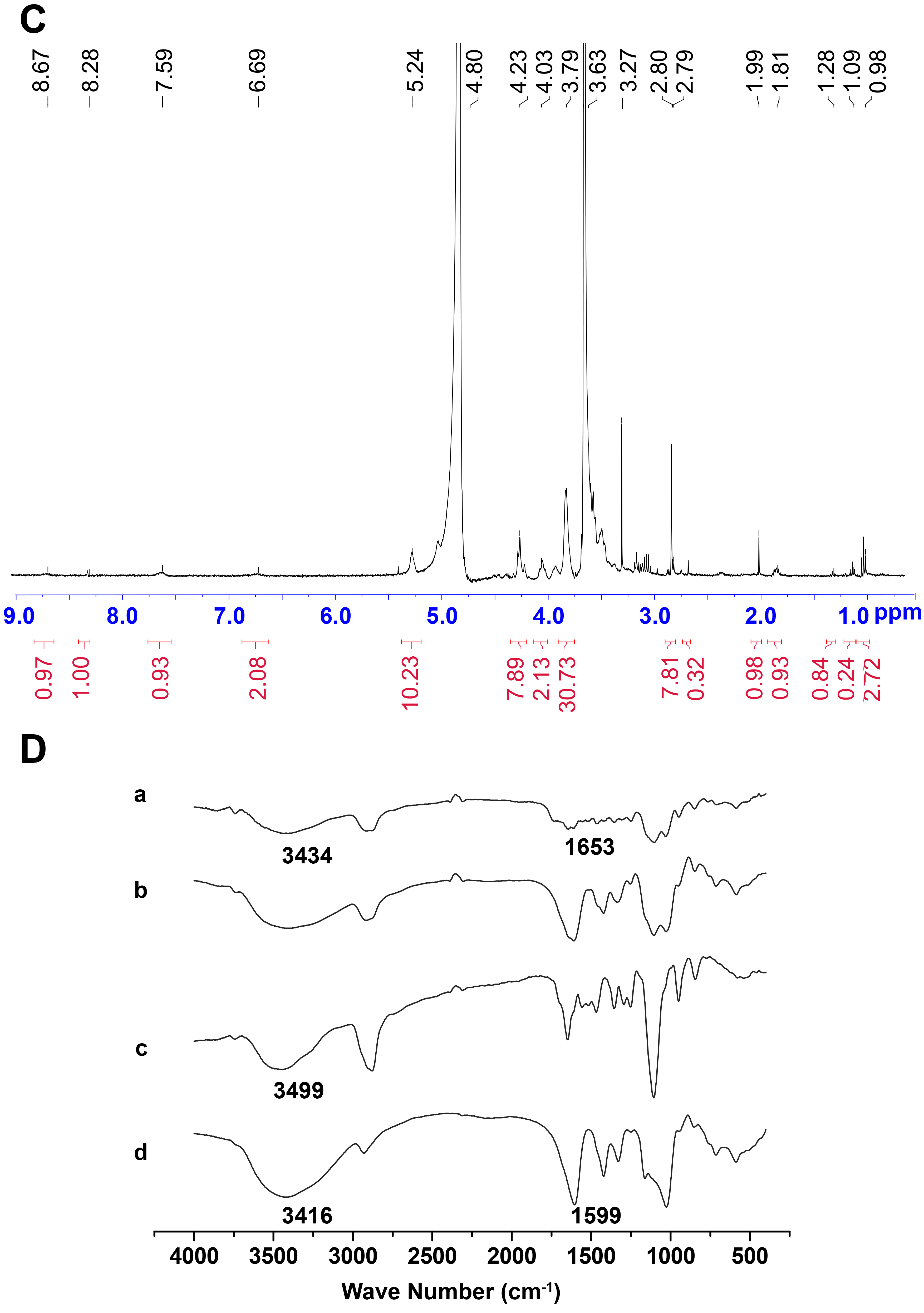


Figure S3. Structural characterization of FA-PEG-CM-β-CD. ^1^H-NMR spectra of CM-β-CD (A), FA-PEG-NH_2_ (B), and FA-PEG-CM-β-CD (C). Fourier transform infrared spectra (D) of FA-PEG-CM-β-CD (a), mixture of FA-PEG-NH_2_ and CM-β-CD (b), FA-PEG-NH_2_ (c), and CM-β-CD (d).


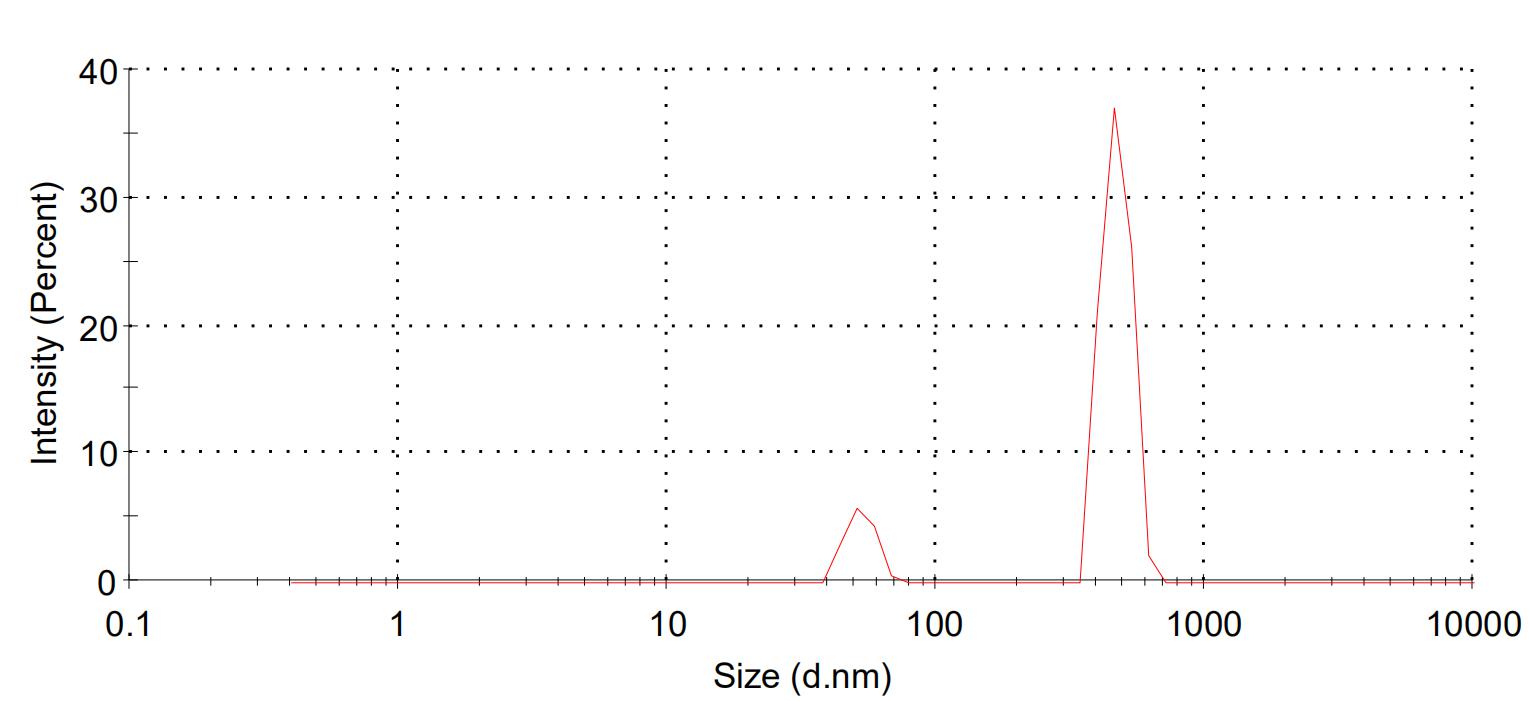


Figure S4. Particle size distribution of BBA/FA-PEG-CM-β-CD.
